# Long noncoding RNA *GATA2-AS1* augments endothelial hypoxia inducible factor 1-α induction and regulates hypoxic signaling

**DOI:** 10.1016/j.jbc.2023.103029

**Published:** 2023-02-17

**Authors:** H.S. Jeffrey Man, Noeline Subramaniam, Tiana Downs, Aravin N. Sukumar, Aninda D. Saha, Ranju Nair, Lucy Chen, Daniel Teitelbaum, Paul J. Turgeon, Kyung Ha Ku, Eileen Tran, Marc de Perrot, Philip A. Marsden

**Affiliations:** 1 Temerty Faculty of Medicine, Institute of Medical Science, University of Toronto, Toronto, Ontario, Canada; 2 Keenan Research Centre in the Li Ka Shing Knowledge Institute, St Michael’s Hospital, University of Toronto, Toronto, Ontario, Canada; 3 Latner Thoracic Surgery Research Laboratories, Toronto General Hospital Research Institute, University of Toronto, Toronto, Ontario, Canada; 4 Division of Respirology, Department of Medicine, University Health Network, University of Toronto, Toronto, Ontario, Canada; 5 Department of Medical Biophysics, University of Toronto, Toronto, Ontario, Canada; 6 Department of Laboratory Medicine and Pathobiology, University of Toronto, Toronto, Ontario, Canada; 7 Division of Thoracic Surgery, Toronto General Hospital, Toronto, Ontario, Canada; 8 Division of Nephrology, Department of Medicine, University of Toronto, Toronto, Ontario, Canada

**Keywords:** endothelial cell, vascular biology, hypoxia, angiogenesis, long noncoding RNA (lncRNA)

## Abstract

Vascular endothelial cells form the inner cellular lining of blood vessels and have myriad physiologic functions including angiogenesis and response to hypoxia. We recently identified a set of endothelial cell (EC)-enriched long noncoding RNAs (lncRNAs) in differentiated human primary cell types and described the role of the STEEL lncRNA in angiogenic patterning. We sought to further understand the role of EC-enriched lncRNAs in physiologic adaptation of the vascular endothelium. In this work, we describe an abundant, cytoplasmic, and EC-enriched lncRNA, *GATA2-AS1*, that is divergently transcribed from the EC-enriched developmental regulator, GATA2. While *GATA2-AS1* is largely coexpressed with GATA2 in ECs, *GATA2-AS1* and GATA2 appear to be complementary rather than synergistic as they have mostly distinct target genes. Common single nucleotide variants in *GATA2-AS1* exons are associated with early-onset coronary artery disease and decreased expression of *GATA2-AS1* in endothelial cell lines. In most cells, HIF1-α is central to the transcriptional response to hypoxia, while in ECs, both HIF1-α and HIF2-α are required to coordinate an acute and chronic response, respectively. In this setting, *GATA2-AS1* contributes to the “HIF switch” and augments HIF1-α induction in acute hypoxia to regulate HIF1-α/HIF2-α balance. In hypoxia, *GATA2-AS1* orchestrates HIF1-α-dependent induction of the glycolytic pathway and HIF1-α-independent maintenance of mitochondrial biogenesis. Similarly, *GATA2-AS1* coordinates both metabolism and “tip/stalk” cell signaling to regulate angiogenesis in hypoxic ECs. Furthermore, we find that *GATA2-AS1* expression patterns are perturbed in atherosclerotic disease. Together, these results define a role for *GATA2-AS1* in the EC-specific response to hypoxia.

Long noncoding RNAs (lncRNAs) that have little to no protein-coding capacity have emerged as major features of eukaryotic genomes and important regulators of development, homeostasis, and disease (1, 2, 3, 4, 5). lncRNAs are loosely defined as RNA transcripts longer than 200 nucleotides that are not translated into protein (6). Current catalogues suggest that there are 17,944 to 27,919 lncRNAs in the human genome (7, 8) that are often expressed in a tissue-specific manner (9, 10, 11, 12).

Several lncRNAs have now been described in vascular endothelial cells (ECs) and have roles in endothelial gene regulation and physiologic function (6). These include modulation of tip and stalk cell biology in angiogenesis (13, 14, 15). Other lncRNAs are responsive to changes in environmental conditions such as hypoxia and shear stress (13, 14, 15, 16).

We recently identified a set of EC-enriched lncRNAs and described the role of the *STEEL* lncRNA in angiogenic patterning (15). *STEEL* is expressed from the HOX loci, which specify regional identities in development (17), and ties EC angiogenic potential to shear stress conditions (15).

To further elucidate the role of EC-enriched lncRNAs in EC biology, we describe *GATA2-AS1*, an EC-enriched lncRNA expressed from the *GATA2* genomic locus. GATA2 is one of six GATA transcription factors and is critical for hematopoiesis (18, 19, 20, 21) and differentiation of endothelial and related lineages (22). Of note, single nucleotide variants (SNVs) at this locus are associated with early-onset coronary artery disease (CAD) and lower expression of *GATA2-AS1*. We find that *GATA2-AS1* modulates the EC hypoxic response by augmenting HIF1-α protein expression and thus affecting HIF1-α/HIF2-α balance. In this way, *GATA2-AS1* sets the stage for an EC-specific response to hypoxia. Importantly, we find disrupted expression patterns of *GATA2-AS1* in vascular disease.

## Results

### *GATA2-AS1* is an endothelial-enriched, abundant lncRNA

**Figure 1:**
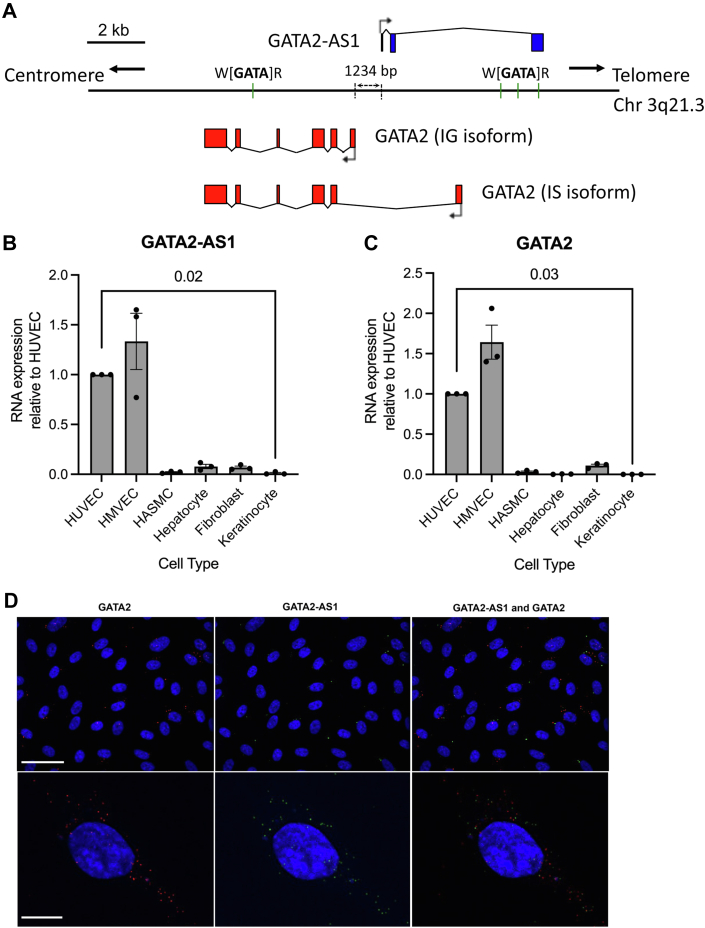
***GATA2-AS1* is an endothelial-enriched, predominantly cytoplasmic long noncoding RNA expressed from the *GATA2* locus.***A*, schematic representation of the *GATA2-AS1* locus. *GATA2-AS1* is transcribed antisense to *GATA2*. There are 1234 bp separating the transcription start site of *GATA2-AS1* from the *GATA2* IG promoter. There is no exon–exon overlap between *GATA2-AS1* and *GATA2* variants. GATA binding sites (W[GATA]R) are depicted as green bars. *B* and *C*, real-time quantitative PCR showing endothelial cell–enriched expression of *GATA2-AS1* (*B*) and *GATA2* (*C*). Kruskal–Wallis test, SEM, n =3. *D*, single molecule RNA-FISH of *GATA2* (*red*) and *GATA2-AS1* (*green*) in HUVECs. *Top panels* show *GATA2* and *GATA2-AS1* expression at 20× magnification. The *top* scale bar represents 50 μm. Colocalization is shown in the last column. *Bottom panels* show higher-resolution images focused on a single cell at 63× magnification. Both *GATA2* and *GATA2-AS1* RNA can be seen in the cytoplasm and at high abundance in a single cell. The *bottom* scale bar represents 10 μm. Images taken with Spinning Quorum Disc Confocal Microscope with a 20× objective with 0.6 numerical aperture and 63× oil immersion objective with 1.4 numerical aperture at room temperature and an EM-CCD camera (Hamamatsu ImageEMX2). Stains used were DAPI (405), Green/*GATA2-AS1* (Alexa 488), Red/*GATA2* (Cy3-555). Images were analyzed in Imaris Image Analysis Software and FIJI. HASMC, human aortic smooth muscle cell; HMVEC, human dermal microvascular endothelial cell; HUVEC, human umbilical vein endothelial cell.

This study examines the lncRNA *GATA2-AS1* (NCBI RefSeq NR_125398.1, Gencode Gene ENSG00000244300.2), which is transcribed antisense and divergently to the transcription factor GATA2 (Fig. 1*A*). The *GATA2-AS1* RNA (Encode transcript *ENST00000464242.1*) is 1163 nt with three exons, and the transcription start site (TSS) is 1234 bp from the *GATA2* TSS. Over the course of this study an alternative splice isoform of *GATA2-AS1* with 1578 nt and two exons was annotated in Gencode v29 (*ENST00000468377.1*). These isoforms share the TSS, but the first splice site is absent in the longer isoform. There are syntenic orthologs of *GATA2-AS1* in other species.

Both *GATA2* and *GATA2-AS1* are EC-enriched and expressed across a variety of EC types (Figs. 1, *B* and *C* and S1, *A*–*C*) (23). *GATA2-AS1* is expressed across a variety of tissues with the highest expression in lung, placenta, and kidney, which are all highly vascularized (Fig. S2*A*). Single molecule RNA FISH (smRNA-FISH) and absolute copy number analysis by Real-time quantitative PCR (RT-qPCR) in human umbilical vein endothelial cells (HUVECs) show that *GATA2-AS1* is relatively abundant at ∼80 to 100 copies per cell, approximately half the expression of *GATA2* mRNA (Fig. S3, *A* and *B*). In comparison, the endothelial-enriched and predominantly nuclear lncRNA, *STEEL*, and the cytoplasmic lncRNA, *H19*, have copy numbers per cell <5 (15, 24). *GATA2-AS1* and *GATA2* are the most highly expressed GATA loci in cultured HUVECs (Fig. S2*B*).

Despite the absence of a canonical polyadenylation sequence, *GATA2-AS1* is predominantly in the polyA+ fraction (Fig. S3*A*). smRNA-FISH (Figs. 1*D* and S3*B*) and subcellular fractionation (Fig. S4*B*) demonstrated predominant cytoplasmic localization of *GATA2-AS1*.

BLASTX (25), ATGpr (26) and the coding potential assessment tool (27) (Fig. S4*C*) all showed that *GATA2-AS1* is unlikely to code for protein. We could not identify any translated ORFs (longest ORF = 103aa) for *GATA2-AS1* in public databases of ribosome profiling (28, 29).

### Genetic variation of *GATA2-AS1*

**Figure 2:**
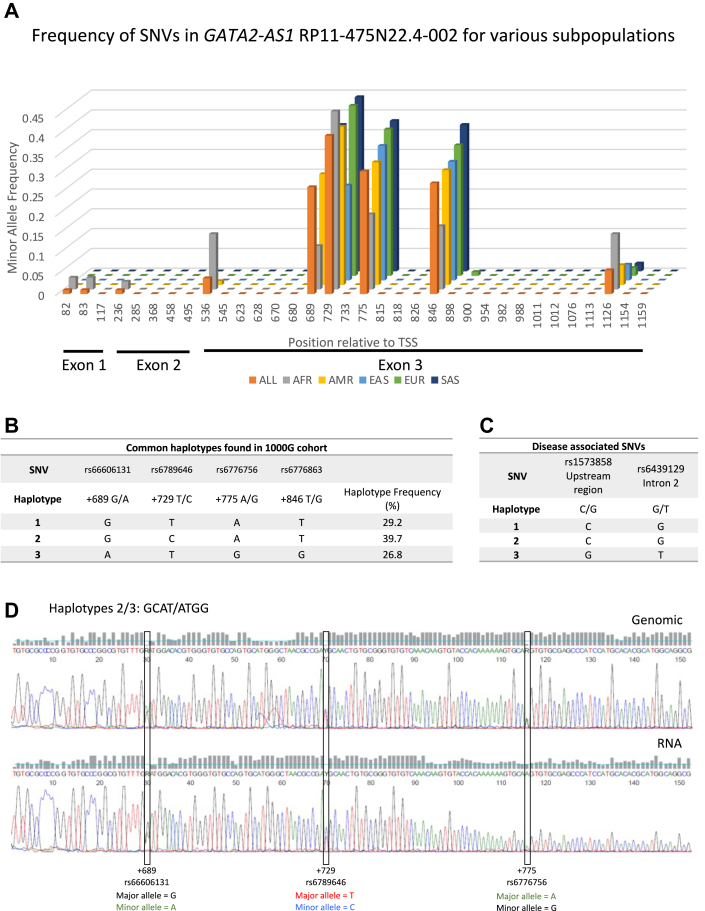
**Genetic variation of *GATA2-AS1* sequences associated with disease and decreased expression.***A*, minor allele frequencies of single nucleotide variants (SNVs) in exons of *GATA2-AS1* from the 1000 genomes project (www.1000genomes.org, https://www.internationalgenome.org accessed March 15, 2020) (Table S4). The position of each SNV relative to the *GATA2-AS1* transcription start site (TSS) is shown. *B*, frequency of common haplotypes of SNVs within *GATA2-AS1* across the 1000 genomes dataset (n = 2504). Three major haplotypes are seen. Four SNVs within *GATA2-AS1* exons are indicated with their positions *versus* the TSS (+689, +729, +775, +846). *C*, SNVs within the *GATA2-AS1* upstream region (rs1573858) and intron 2 (rs6439129) that have been associated with disease susceptibility for early-onset coronary artery disease are shown. These disease-associated SNVs are in linkage disequilibrium with SNVs within *GATA2-AS1* exons. Disease alleles are associated with Haplotype 3. *D*, sequencing of genomic DNA and RNA from human umbilical vein endothelial cells (HUVECs). Example of a heterozygote for haplotype 2/3 is shown. The genomic sequencing (*top*) shows equal peak heights for bases representing both alleles at each SNV site (+689, +729, +775). The RNA sequencing (*bottom*) shows bigger peak heights for bases representing alleles for Haplotype 2 (GCAT) *versus* Haplotype 3 (ATGG) at each SNV site. All three HUVEC lines with these heterozygous haplotypes demonstrated similar results with a 2:1 ratio of sequencing peak heights for Haplotype 1 or Haplotype 2: Haplotype 3. AFR, Africa; AMR, America; EAS, East Asian; EUR, European; SAS, South Asian.

The *GATA2* genomic locus has been identified as conferring susceptibility to human genetic disease (18, 30). Three conserved GATA switch sites proposed to determine *GATA2* expression lie within the *GATA2-AS1* genomic locus (18). At one of these sites, ∼−3.9 kb relative to the endothelial *GATA2* TSS, we found several SNVs with a common (>5%) minor allele frequency (Figs. 2*A* and S5). These sites fall within exon 3 of *GATA2-AS1* but do not involve canonical W[GATA]R GATA-binding sequences. Individuals from Africa harbor the most variant sites, consistent with the out-of-Africa model of human origins (31).

We analyzed all individuals from the 1000 genomes project and found three common (>5%) and three rare (<5%) haplotypes (31), consistent with data from eight independent primary HUVEC lines from our laboratory (Figs. 2*B* and S6, *A*–*C*, Table S4). Positions +689, +775, and +846 (relative to *GATA2-AS1* TSS) appear to segregate together, while position +729 can be variable relative to the other sites. Minor alleles at positions +689, +775, and +846 (haplotype 3) are in linkage disequilibrium with SNVs in upstream genomic regions and intron 2 of *GATA2-AS1* (rs1573858 and rs6439129, respectively) (Fig. 2*C*) that have been associated with susceptibility to early-onset CAD (32).

To assess the effect of SNVs on *GATA2-AS1* expression, we sequenced cDNA from HUVEC cell lines and found a 2:1 ratio of peak heights for the major:minor allele at positions +689, +775, and +846 in heterozygotes, indicating higher expression of the major allele at these positions (haplotypes 1 & 2 *versus* haplotype 3) (Fig. 2*D*). We found no difference in expression between haplotypes 1 and 2 (Fig. S5*D*). Notably, the haplotype that carried the minor allele at all four of these SNVs (haplotype “ACGG”) occurred at a markedly lower frequency than the other haplotypes and had a different predicted secondary structure compared with major haplotypes (Figs. S6*B* and S7) (33). Thus, disease-associated minor alleles within *GATA2-AS1* exon sequences are associated with *GATA2-AS1* expression.

### Evidence for antisense lncRNA expression in *GATA* loci

The six *GATA* genes in vertebrate genomes represent two classes, *GATA123* and *GATA456*, that originated from two ancestral deuterostome genes *via* genome duplication events (34). *GATA6-AS* has been reported as a hypoxia-regulated lncRNA (13). Therefore, we asked whether antisense lncRNA expression was a feature of other *GATA* loci in humans and found that 4/6 *GATA* transcription factors, spanning both *GATA* classes (*GATA123* and *GATA456*), have annotated antisense lncRNAs (Fig. S8). These include *GATA2*, *GATA3*, *GATA5*, and *GATA6*.

Because *GATA* genes evolved *via* duplication events, these findings in the human *GATA* loci could reflect antisense regulatory elements that were also duplicated in evolution. As a result, we examined *GATA* gene loci across vertebrate evolution using existing public databases and looked for lncRNAs within a 5-kb window of *GATA* transcription factors. A *GATA2-AS* lncRNA was conserved in some primates. We found a syntenic ortholog for *GATA2-AS1* in mouse (∼90 million years), with ∼30% RNA sequence conservation in at least one isoform (Fig. S9*A*), consistent with RNA sequence variation of lncRNAs forming an important component of speciation. Overall, we found evidence of antisense lncRNA expression at *GATA* factor loci across ∼300 million years of evolution between humans and chicken (Fig. S9*B*). All *GATA* loci demonstrated expression of an antisense lncRNA in at least one examined species except *GATA1*. However, there does not appear to be any common lineage of *GATA*-antisense lncRNAs between closely related species, in keeping with rapid evolution of lncRNA loci (35). This interspecies variation in lncRNA loci is consistent with intraspecies variation described above.

### *GATA2* and *GATA2-AS1* are generally coexpressed in individual cells

LncRNA expression is often correlated with the expression of nearby genes (36). Single-cell RNA sequencing (scRNAseq) of HUVEC showed that *GATA2-AS1* was expressed in the same clusters as *GATA2* (Fig. S3*C*). We confirmed that *GATA2-AS1* and *GATA2* are coexpressed in the same cells using smRNA-FISH. Overall, ∼39% of cells express *GATA2-AS1* (Fig. 1*D*) and ∼61% of cells express *GATA2*. Of cells expressing *GATA2-AS1*, a majority (∼78%) also express *GATA2*, and of cells that express *GATA2*, half (∼50%) express *GATA2-AS1* (Figs. 1*D* and S3*D*). A similar degree of overlap was noted in scRNAseq of HUVEC, although the absolute fraction of cells expressing *GATA2-AS1* or *GATA2* was lower, possibly reflecting lower sensitivity of scRNAseq compared with smRNA-FISH (37). These results demonstrate that *GATA2-AS1* and *GATA2* expression are not mutually exclusive.

### GATA2 and *GATA2-AS1* have distinct target genes

**Figure 3:**
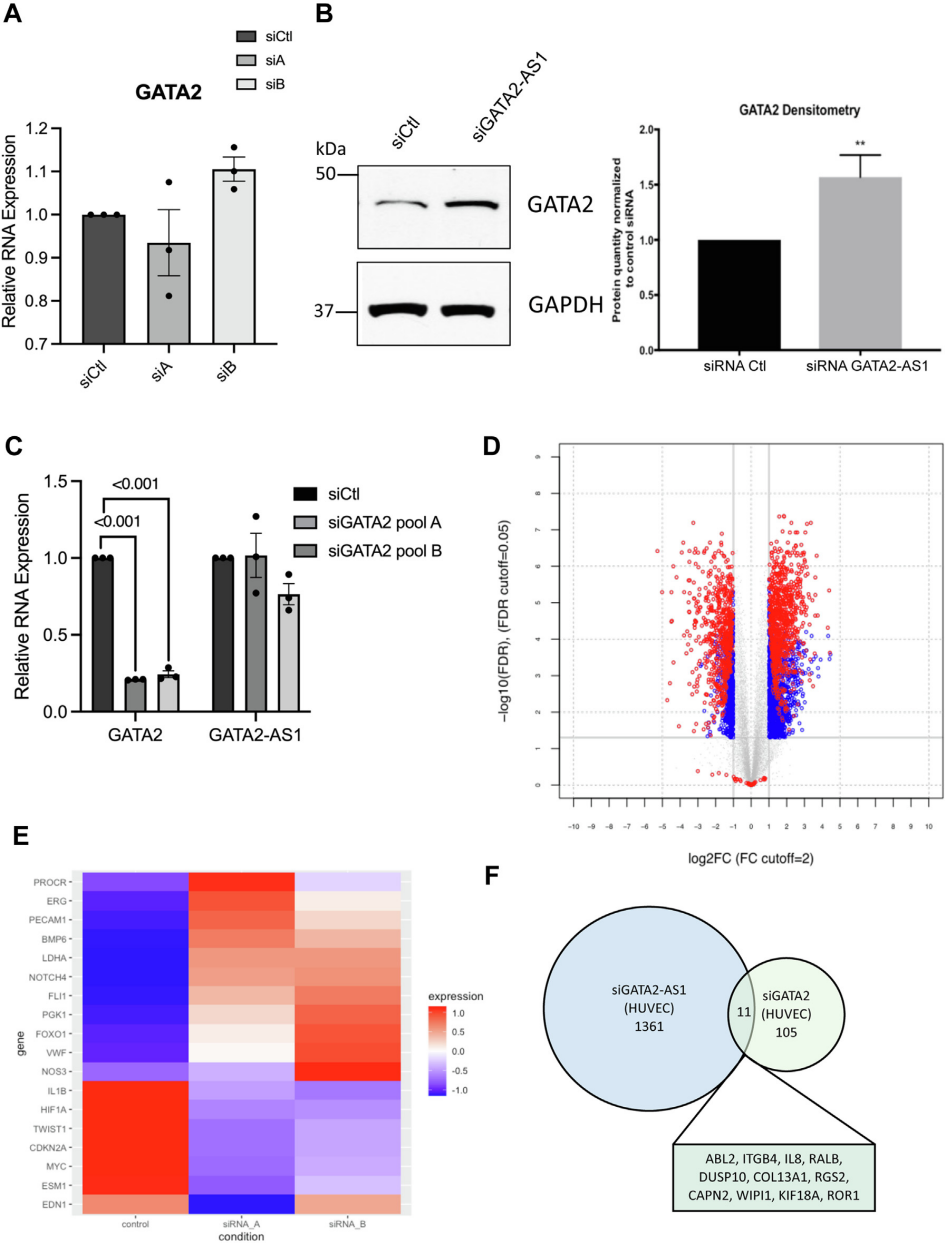
***GATA2-AS1* and GATA2 are functionally distinct.***A*, effect of *GATA2-AS1* knockdown by siRNA on *GATA2* mRNA. Real-time quantitative PCR of *GATA2* mRNA is shown. *B*, effect of *GATA2-AS1* knockdown by siRNA on GATA2 protein levels. *Left*, representative Western blot of GATA2 protein. GAPDH is shown as a loading control. *Right*, quantitation of Western blots showing GATA2 protein levels. *C*, effect of GATA2 knockdown by siRNA on *GATA2-AS1* RNA levels. GATA2 knockdown by siRNA did not significantly alter *GATA2-AS1* RNA expression. One-way ANOVA, SEM, n = 3. Normality tested with Shapiro–Wilks test. *D*, volcano plot showing microarray analysis of differentially regulated mRNAs with *GATA2-AS1* knockdown. Shown are mRNAs differentially regulated by both *GATA2-AS1* siRNAs. A total of 782 protein-coding mRNAs were upregulated, and 592 were downregulated 2-fold or more. Statistical significance was assessed by Student *t* test with a Benjamini–Hochberg adjusted false discovery rate <0.05. *Gray dots* indicate hits that fell below fold change and significance cutoffs. *Blue dots* indicate hits that exceed fold change and significance cutoffs in at least one siRNA, and *red dots* indicate hits that exceed fold change and significance cutoffs with both siRNAs that target *GATA2-AS1*. *E*, heatmap showing microarray data from *GATA2-AS1* knockdown. Select upregulated and downregulated genes are shown. Expression level represents z-score of expression for each gene. *F*, Venn diagram showing overlap of *GATA2-AS1* mRNA target genes and GATA2 target genes (26). In human umbilical vein endothelial cells, *GATA2-AS1* regulated 11/116 (∼9.5%) of GATA2 target genes. Unless otherwise stated, statistical significance was assessed by two-sided Student *t* test and ∗∗*p* < 0.01.

LncRNAs can act *in cis* to regulate neighboring genes (9, 11, 38). To determine if there was regulation of GATA2 expression by the *GATA2-AS1* transcript, we depleted *GATA2-AS1* using siRNA. We found a small decrease in *GATA2* mRNA levels but note an increase in GATA2 protein levels (Figs. 3, *A* and *B* and S10, *A* and *B*). GATA2 is known to bind to genomic regions corresponding to exon 3 of *GATA2-AS1* in ECs (18). To determine an effect of GATA2 on *GATA2-AS1* expression, we used siRNA targeting *GATA2* and found no major effects on *GATA2-AS1* RNA expression (Fig. 3*C*). While these results do not rule out regulation of *GATA2-AS1* by GATA2, GATA2 does not appear to be a critical activator of *GATA2-AS1* in ECs.

A high degree of overlap between *GATA2-AS1* and GATA2 targets genome-wide could also suggest *cis*-regulation or synergy as a major feature of this locus. We used siRNA knockdown of *GATA2-AS1* in HUVEC followed by microarray analysis to assess *GATA2-AS1* targets on a genome-wide scale. Overall, 1374 protein-coding mRNAs and 375 lncRNAs were regulated in common between two siRNA sequences targeting *GATA2-AS1* (Figs. 3, *D* and *E* and S10, *C* and *D*). Of those, 782 protein-coding mRNAs and 166 lncRNAs were upregulated, and 592 and 209 were downregulated, respectively. Gene Ontology analysis revealed *GATA2-AS1* target genes function across a variety of intracellular processes, including response to stress (Fig. S10*D*) (39).

We compared our list of target genes to published GATA2 targets in HUVEC (GEO Accession: GSE29531) (40), which identified 116 GATA2 target genes, and found 11 protein-coding genes in common (Figs. 3*F*, S11 and S12). While some target genes are shared, the majority of *GATA2-AS1* and GATA2 regulated protein-coding genes appear distinct, although technical differences in microarray platform and hybridization may underestimate the overlap in target genes.

### *GATA2-AS1* responds to environmental stimuli

**Figure 4:**
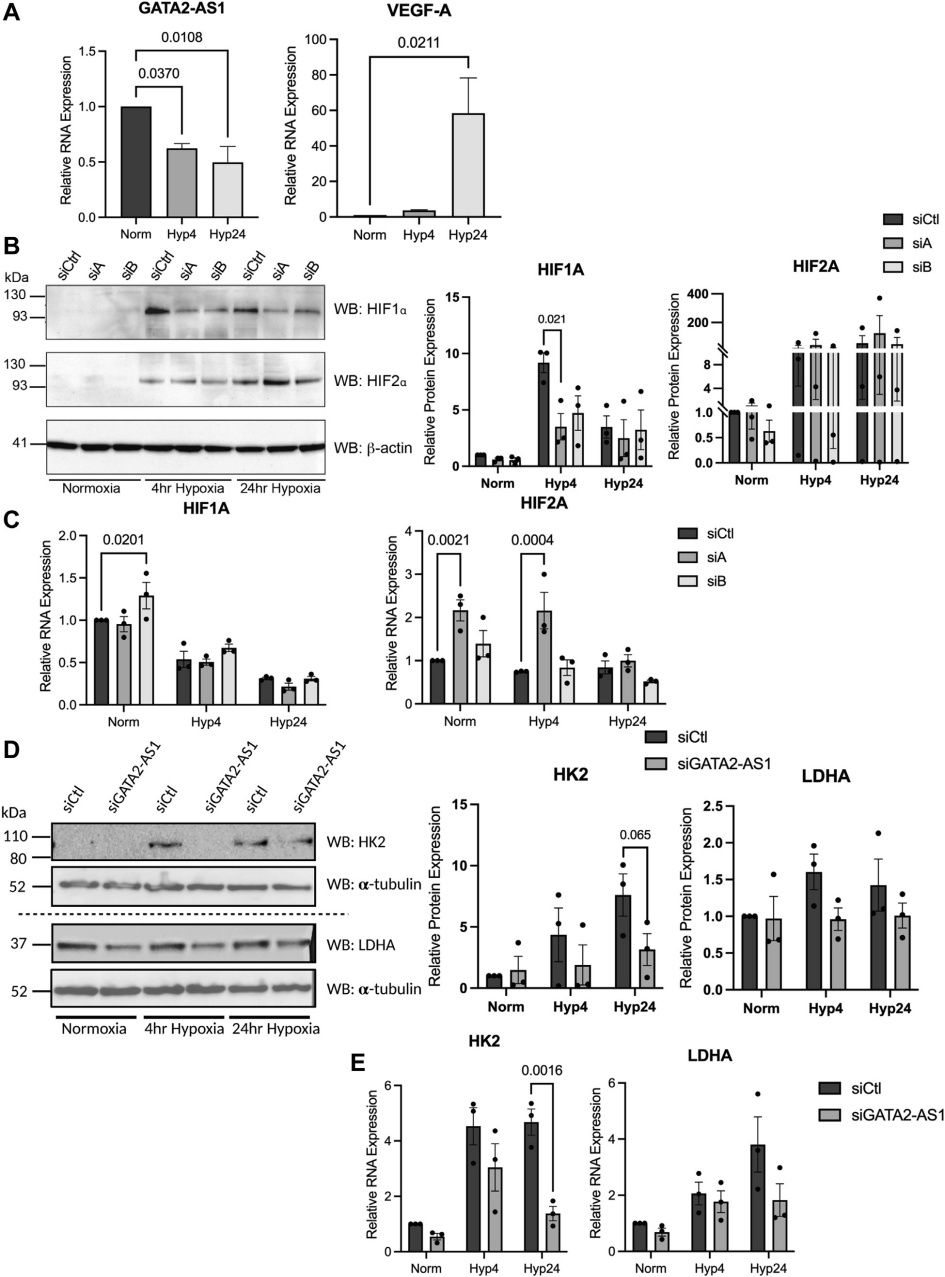
***GATA2-AS1* augments the induction of HIF1-α and metabolic signaling with hypoxia.***A*, RT-qPCR of *GATA2-AS1* and *VEGFA* in hypoxia. *B*, *left*, representative Western blots for HIF1-α and HIF2-α with *GATA2-AS1* siRNA knockdown in normoxia, acute (4 h) and chronic (24 h) hypoxia. β-Actin is used a loading control. *Middle*, quantitation of Western blots for HIF1-α show that *GATA2-AS1* knockdown decreases HIF1-α induction in acute (4 h) hypoxia. *Right*, quantitation of HIF2-α protein shows no consistent change in HIF2-α levels with *GATA2-AS1* knockdown. *C*, RT-qPCR of HIF1A (*left*) and *EPAS1*/HIF2A (*right*) with *GATA2-AS1* knockdown in acute and chronic hypoxia. *D*, *left*, representative Western blots of HK2 and LDHA with *GATA2-AS1* siRNA knockdown in normoxia, acute (4 h) and chronic (24 h) hypoxia. α-Tubulin is shown as a loading control; loading control for HK2 corresponds to the loading control for BNIP3L (Fig. 5) and loading control for LDHA corresponds to the loading control for BNIP3 (Fig. 5). Consistent with mRNA levels, there is a decrease in HK2 and LDHA protein with siGATA2-AS1, especially in hypoxia. Middle, QUANTITATION of Western blots for HK2 show that *GATA2-AS1* siRNA knockdown decreases HK2 induction in chronic (24 h) hypoxia. *Right*, quantitation of LDHA protein shows trend toward a decrease in LDHA levels with *GATA2-AS1* knockdown in acute and chronic hypoxia. *E*, RT-qPCR of *HK2* and *LDHA* with *GATA2-AS1* knockdown in acute and chronic hypoxia. Statistical significance between siCtl and siGATA2-AS1 was assessed by two-way ANOVA, SEM, n = 3. Normality tested by Shapiro–Wilks test. RT-qPCR, real-time quantitative PCR.

Promoter analysis (−2000 → +1 relative to TSS) found putative elements for hypoxia, shear stress, and NF-κB (Fig. S13, *A*–*C*). Consistent with *GATA6-AS*, *GATA2-AS1* was also found to be hypoxia responsive. We found downregulation of *GATA2-AS1* at 24 h of hypoxia (1%) (Figs. 4*A* and S13*D*). In HUVEC, KLF2 overexpression increases *GATA2-AS1* expression by Nanostring counts (Fig. S13*E*). *GATA2-AS1* decreases with hypoxia and laminar flow in arterial-like and venous-like ECs derived from hPSCs (Fig. S13*F*) (41, 42).

### *GATA2-AS1* modulates HIF1-α induction in acute hypoxia

Microarray in HUVEC showed that *HIF1A* mRNA was downregulated with *GATA2-AS1* knockdown in normoxia (Fig. 3*D*). However, HIF biology is regulated at the level of the protein, whereby HIF-α proteins are constitutively expressed and stable in the absence of oxygen but degraded in the presence of oxygen (43, 44). Accordingly, we sought to assess the regulation of HIF1-α protein by *GATA2-AS1* under hypoxic conditions. We depleted *GATA2-AS1* RNA in normoxia and acute (4 h) and chronic (24 h) hypoxia (<1% O_2_). *GATA2-AS1* knockdown decreased HIF1-α protein induction with acute hypoxia (Fig. 4*A*). HIF1-α levels were unaffected by *GATA2-AS1* knockdown with chronic hypoxia. Two factors may contribute to this observation. First, HIF1-α protein expression is lower with 24 h *versus* 4 h hypoxia, consistent with the literature (45). Second, *GATA2-AS1* RNA levels are already decreased with 24 h of hypoxia (Fig. 4*A*). Nonetheless, this effect appears to be specific to HIF1-α as HIF2-α protein induction did not decrease with *GATA2-AS1* knockdown.

Consistent with previous data (46), we found a decrease in *HIF1A* but not *EPAS1*/*HIF2A* mRNA with acute and chronic hypoxia. Knockdown of *GATA2-AS1* with hypoxia did not significantly change *HIF1A* or *HIF2A* mRNA levels and suggests posttranscriptional regulation of HIF1-α protein levels, consistent with the cytoplasmic localization of *GATA2-AS1* RNA (Fig. S14, *A*–*C*). As reviewed by us and others, the cytoplasmic effects of lncRNAs are protean, so in future, the mechanism of *GATA2-AS1* effects in acute hypoxia will be of great interest (47). Overexpression of *GATA2-AS1* did not increase HIF1-α protein levels in acute hypoxia, suggesting a threshold effect. However, there was a small but variable increase in HIF1-α protein levels by 1.6-fold relative to control with chronic hypoxia when *GATA2-AS1* and HIF1-α protein levels are normally reduced (Fig. S15, *A* and *E*). Thus, *GATA2-AS1* overexpression maintained HIF1-α protein levels in chronic hypoxia at a similar level to acute hypoxia. In summary, we find a major effect of *GATA2-AS1* in priming acute HIF1-α protein induction in response to acute hypoxia.

### *GATA2-AS1* maintains the expression of glycolytic pathway genes in hypoxia

In hypoxia, ECs demonstrate increased anaerobic glucose metabolism through increased expression of HIF1-α target genes (48) such as *HK1* and *LDH* (49). Indeed, *GATA2-AS1* knockdown with hypoxia decreased many HIF1-α-specific target genes in the glycolytic pathway, such as *HK1*, *HK2*, *LDHA*, *PGK1*, and *MCT4* (Figs. 4, *B* and *C* and S14, *E*–*H*). The HIF2-α response is an organismal response, and characterized by *EPO*, which increased with *GATA2-AS1* knockdown. A greater decrease in mRNA levels of target genes at 24 h hypoxia may result from persistent mRNA at 4 h hypoxia due to mRNA half-life despite a decrease in HIF1-α-mediated transcription. These results suggest a role for *GATA2-AS1* in regulating HIF1-α-mediated metabolic reprogramming in response to hypoxia and the balance of HIF1-α and HIF2-α signaling.

### *GATA2-AS1* maintains mitochondrial mass in hypoxia

**Figure 5:**
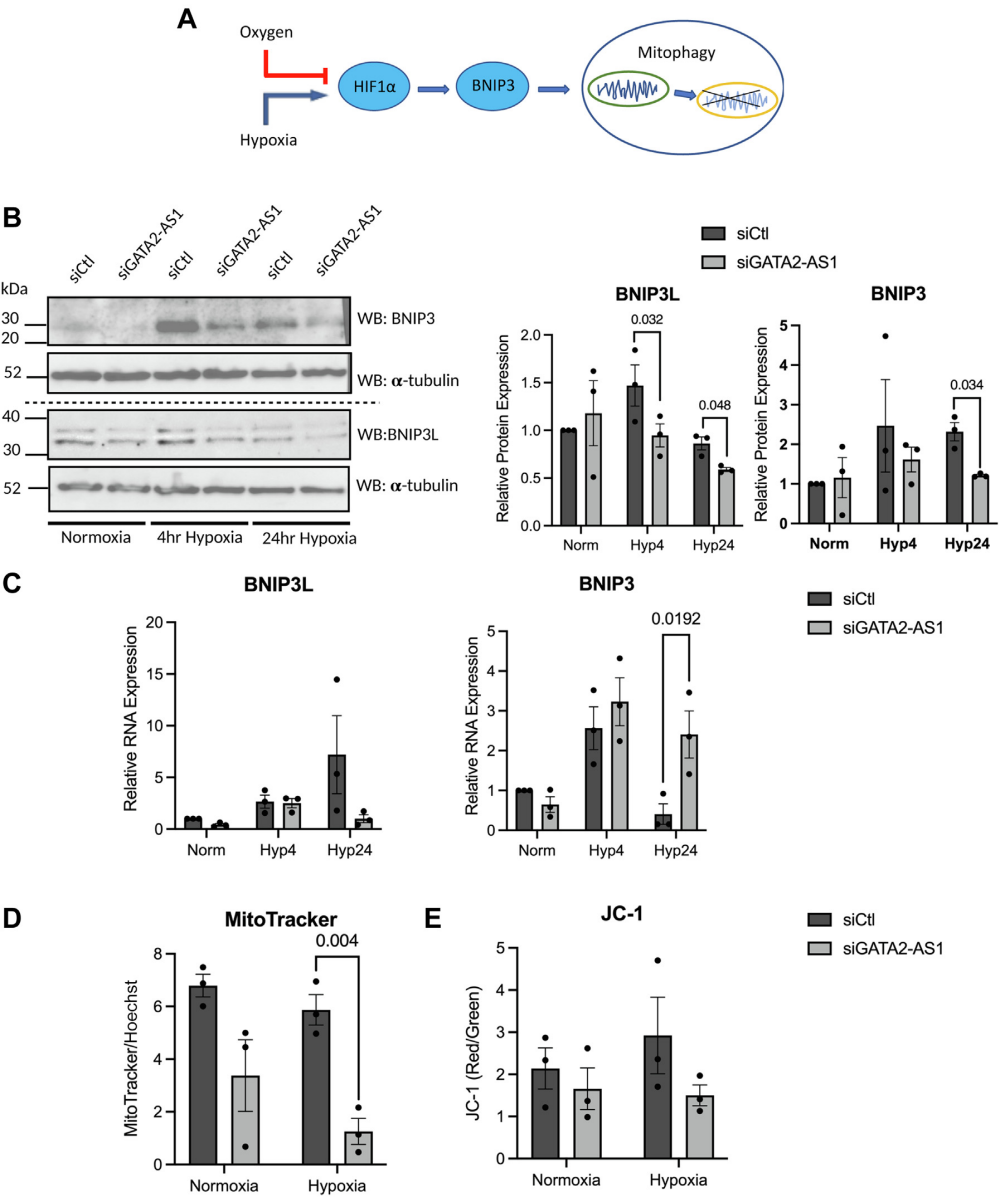
***GATA2-AS1* maintains mitochondrial mass in hypoxia.***A*, schematic showing expected relationship of hypoxia with mitochondrial dynamics through HIF signaling. *B*, *left*, representative Western blot of BNIP3L and BNIP3 with *GATA2-AS1* knockdown in normoxia and hypoxia. BNIP3L and BNIP3 are HIF1-α-specific target genes. α-Tubulin is shown as a loading control; loading control for BNIP3 corresponds to loading control for LDHA (Fig. 4) and loading control for BNIP3L corresponds to loading control for HK2 (Fig. 4). There is a decrease in BNIP3L and BNIP3 protein with *GATA2-AS1* knockdown in hypoxia. *Middle*, quantitation of Western blots for HIF1-α show that *GATA2-AS1* siRNA knockdown decreases BNIP3L induction in hypoxia. *Right*, quantitation of BNIP3 protein shows that *GATA2-AS1* knockdown decreases BNIP3 induction in hypoxia. *C*, real-time quantitative PCR of *BNIP3L* (*left*) and *BNIP3* (*right*) with *GATA2-AS1* knockdown in acute and chronic hypoxia. *D*, fluorescence measurements of Mitotracker Green FM in human umbilical vein endothelial cells with *GATA2-AS1* knockdown in normoxia and 24 h hypoxia. Mitotracker tracks mitochondrial abundance independent of membrane potential. Hoechst staining was used to normalize for cell number. There was a decrease in Mitotracker *Green* FM staining with *GATA2-AS1* knockdown, especially in hypoxia. *E*, fluorescence measurements of JC-1 staining with *GATA2-AS1* knockdown in normoxia and 24 h hypoxia. JC-1 staining reflects mitochondrial membrane potential and is represented as a *red*/*green* ratio. JC-1 fluoresces *green* in isolation but aggregates and fluoresces *red* in the presence of negative charge. A higher *red*/*green* ratio indicates presence of mitochondrial membrane potential. There was a trend toward a decrease in mitochondrial membrane potential with *GATA2-AS1* knockdown, especially with hypoxia. Statistical significance between siCtl and siGATA2-AS1 was assessed by two-way ANOVA or two-sided *t* test, SEM, n = 3. Normality tested by Shapiro–Wilks test.

A decrease in oxidative phosphorylation and increased mitophagy is a HIF1-α-dependent event in hypoxia, in part through increased expression of BNIP3 (Fig. 5*A*) (50). In ECs, although there is a decrease in mitochondrial respiration with hypoxia, there is no change in mitochondrial biogenesis (49). Although ECs are highly glycolytic even in the presence of oxygen, mitochondria act as important signaling organelles in ECs (51, 52), including the stabilization of HIF (43, 53). As a result, we assessed the role of *GATA2-AS1* on mitochondrial dynamics. Although we found variable effects of *GATA2-AS1* knockdown on *BNIP3* and *BNIP3L* mRNA in hypoxia (Fig. 5, *B* and *C*), we found a decrease of both BNIP3 and BNIP3L protein expression in acute and chronic hypoxia. As a result, we expected that *GATA2-AS1* knockdown would lead to an increase in mitochondrial mass and function in hypoxic conditions. Surprisingly, *GATA2-AS1* knockdown led to a 50% decrease in mitochondrial mass and a trend toward decreased mitochondrial membrane potential (Fig. 5, *D* and *E*) with 24 h hypoxia. Thus, *GATA2-AS1* contributes to the maintenance of mitochondrial mass in hypoxia, likely through HIF1-α-independent mechanisms.

### *GATA2-AS1* modulates angiogenic patterning and signaling in hypoxia

**Figure 6:**
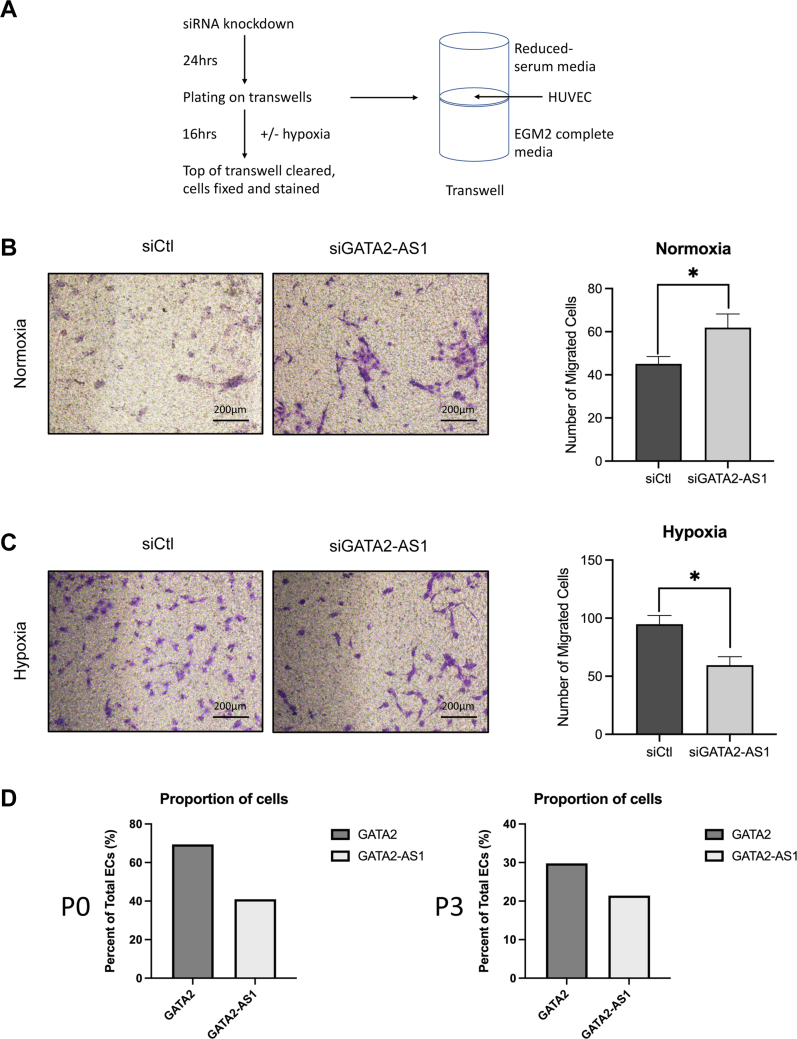
***GATA2-AS1* increases endothelial cell migration in hypoxia.***A*, schematic representation of Transwell migration assay. Images were acquired on a Nikon Eclipse TS100 with a 20× objective and a Nikon Digital Sight camera. *B*, images and quantitation of Transwell migration of human umbilical vein endothelial cells (HUVEC) with *GATA2-AS1* knockdown in normoxia. *GATA2-AS1* knockdown led to an increase in Transwell migration in normoxia. Statistical significance was assessed by two-sided *t* test, SEM, n = 2 biological replicates, n = 9 technical replicates. Normality tested by Shapiro–Wilks test. *C*, images and quantitation of Transwell migration of HUVEC with *GATA2-AS1* knockdown in hypoxia. *GATA2-AS1* knockdown led to a decrease in Transwell migration in hypoxia. A minimum of nine wells were assessed for each condition with six images taken per well. *D*, single-cell RNA-sequencing data of *GATA2-AS1* and *GATA2* expression. In freshly isolated HUVEC (Passage 0 = P0), *GATA2* was expressed in 69% of cells and *GATA2-AS1* was expressed in 41% of cells. In Passage 3 (P3) HUVEC, *GATA2* was expressed in 30% of cells, *GATA2-AS1* was expressed in 21% of cells. For technical reasons, functional studies of *GATA2-AS1* were performed in passaged HUVEC. Unless otherwise stated, statistical significance was assessed by two-sided Student *t* test and ∗ *p* < 0.05.

**Figure 7:**
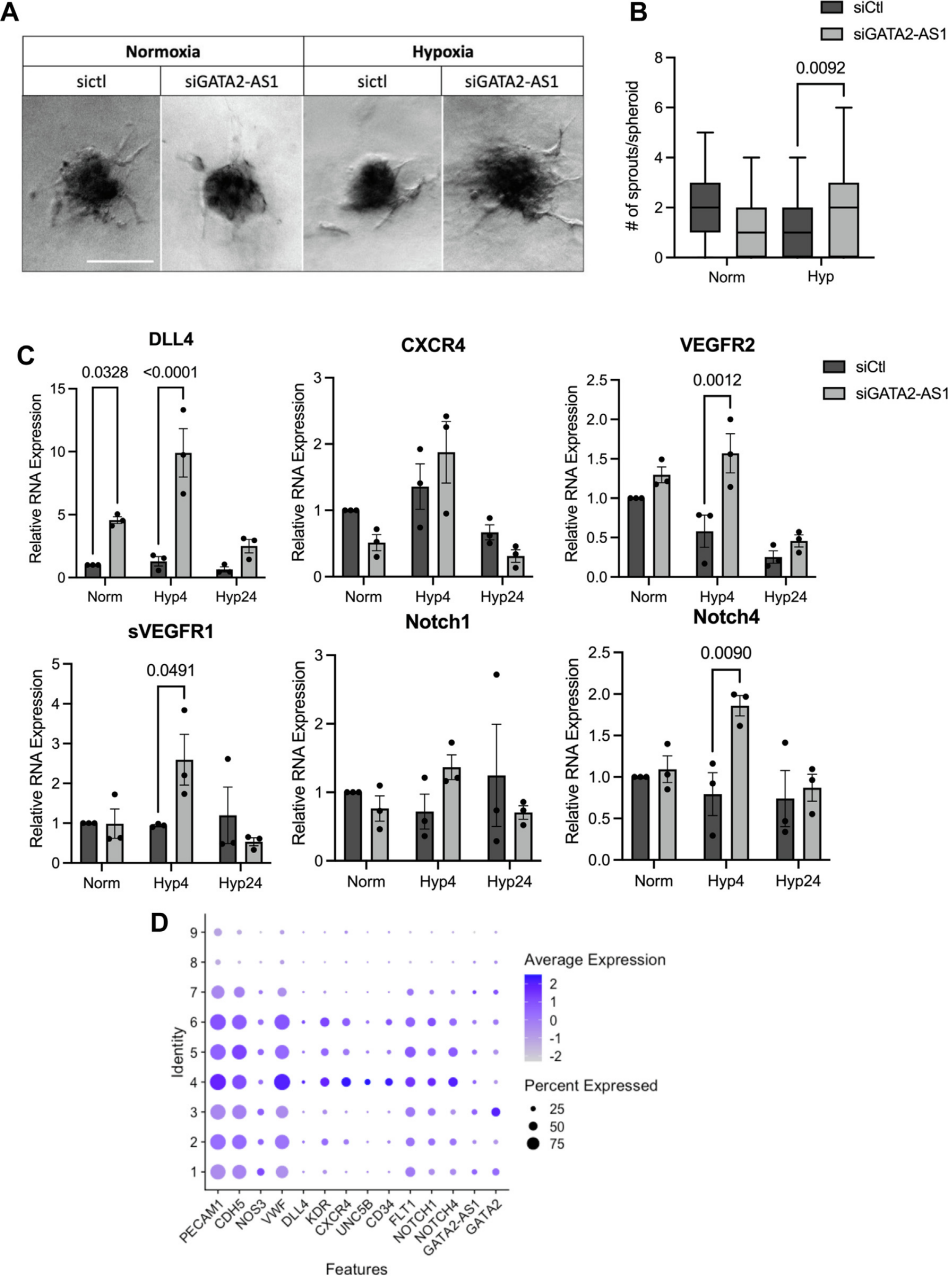
***GATA2-AS1* increases endothelial sprouting in hypoxia.***A*, representative images from spheroid sprouting assay in human umbilical vein endothelial cells (HUVEC) with *GATA2-AS1* knockdown in normoxia and hypoxia. The scale bar represents 100 μm. *B*, quantitation of spheroid sprouting assay (n = 50–100 spheroids per condition) with *GATA2-AS1* knockdown in normoxia and hypoxia. There was an increase in the number of sprouts with GATA-2AS1 knockdown in hypoxia. Kruskal–Wallis test, n = 3. Videos of sprouting assay in normoxia can be seen in Video S1 and S2. Images were acquired on a Zeiss AxioObserver Live Cell with FLIM with a 20× objective at 37 °C and analyzed with FIJI. *C*, real-time quantitative PCR of “tip” and “stalk” cell markers during angiogenesis. *GATA2-AS1* knockdown led to increased expression of both “tip” and “stalk” cell genes in acute (4 h) hypoxia. Statistical significance between siCtl and siGATA2-AS1 was assessed by two-way ANOVA, SEM, n = 3. *D*, single-cell RNA sequencing of cultured HUVEC. Cultured HUVEC display heterogenous gene expression patterns with nine different clusters. Clusters 4 to 6 are associated with increased expression of tip cell genes. Stalk cell genes are expressed across clusters 1 to 7. *GATA2-AS1* is most highly enriched in clusters 1 and 3 but is also expressed in clusters 1 to 7.

Sprouting angiogenesis expands the vascular network in hypoxic tissues. HIF1-α and HIF2-α differentially regulate vessel growth with HIF1-α promoting EC migration and HIF2-α promoting vessel integrity (48, 54, 55, 56). In keeping with these effects on HIF1-α, *GATA2-AS1* knockdown led to decreased Transwell migration of HUVEC in hypoxia (Figs. 6, *A*–*C* and S16, *A*–*C*). In the tip/stalk paradigm of angiogenesis (57), tip cells rely more on anaerobic glycolysis while stalk cells use fatty acid oxidation (51). Therefore, we expected *GATA2-AS1* knockdown to decrease tip cell balance in hypoxia due to its effect on HIF1-α and glycolytic genes. Unexpectedly, we found that *GATA2-AS1* knockdown in chronic hypoxia increased the number of sprouts/spheroid in a sprouting assay (Fig. 7, *A* and *B*). Thus, the effect of *GATA2-AS1* on spheroid sprouting in hypoxia does not appear to be purely dependent on its effect on HIF1-α signaling. To further investigate the role of *GATA2-AS1* on sprouting, we assessed tip and stalk cell gene expression by RT-qPCR. *GATA2-AS1* knockdown in hypoxia led to an increase in tip cell genes such as *DLL4* and *KDR*/*VEGFR2*, as well as stalk cell genes such as *NOTCH1* and *sFLT1*/*sVEGFR1* (51, 58), consistent with the results of the sprouting assay (Fig. 7*C*). These findings suggest both HIF1-α-dependent and HIF1-α-independent effects of *GATA2-AS1* in hypoxic signaling and demonstrate a higher-order role for *GATA2-AS1* in orchestrating the EC-specific response to hypoxia.

**Figure 8:**
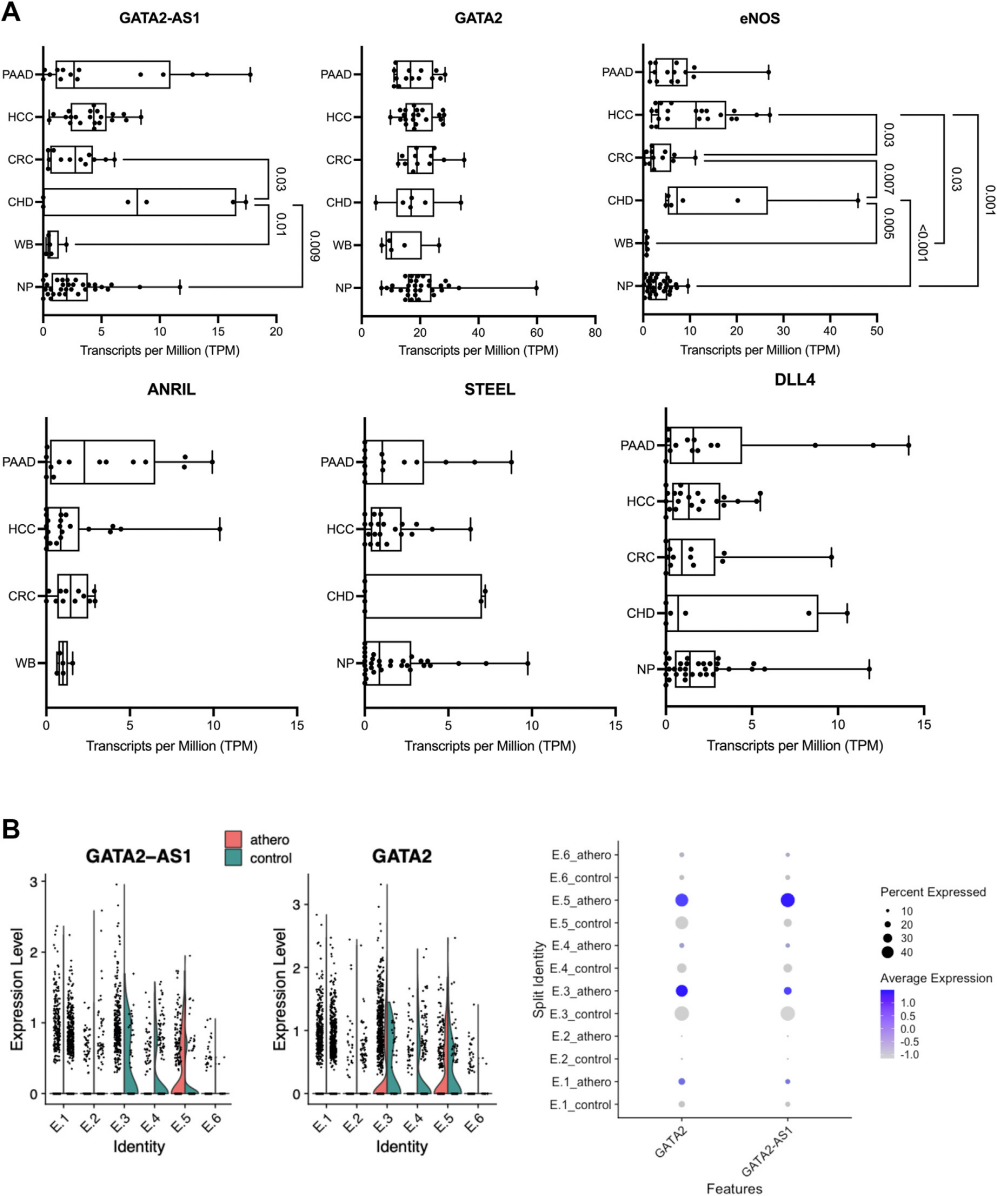
***GATA2-AS1* is differentially expressed in human disease samples.***A*, boxplots of data from exoRBase (http://www.exorbase.org accessed June 23, 2021) show increased expression of *GATA2-AS1* in exosomes of patients with coronary heart disease (CHD) compared with two control groups, healthy controls (Healthy) and whole blood (WB). For comparison *GATA2* mRNA expression is shown. There was no significant difference in *GATA2* mRNA expression in exosomes across these samples. Other endothelial-enriched genes (*NOS3*/eNOS, *STEEL*, *DLL4*) and the *ANRIL* lncRNA are shown for comparison. *B*, dot plot and violin plots from single-cell RNA sequencing of human atherosclerosis tissue show differential expression of *GATA2-AS1* (*left*) and *GATA2* (*right*) in endothelial cell clusters (E.1–E.6). In clusters E.3 and E.4 there is greater expression of *GATA2-AS1* in control tissue compared with atherosclerosis core, and in E.5, there is greater expression of *GATA2-AS1* in atherosclerosis core. Data were accessed from GEO (GSE159677) control, proximal adjacent tissue from the carotid artery. Athero, atherosclerosis core; CHD, coronary heart disease; CRC, colorectal cancer; HCC, hepatocellular carcinoma; PAAD, pancreatic adenocarcinoma; WB, whole blood Unless otherwise stated, statistical significance was assessed by two-sided Student *t* test.

Because *GATA2-AS1* is heterogeneously expressed in ECs (Figs. 6*D* and S3, *C*–*E*), we looked for coexpression of *GATA2-AS1* with tip and stalk cell genes in normoxia with scRNAseq (Fig. 7*D*). *GATA2-AS1* expression is enriched in clusters 1 to 3, whereas tip and stalk cell genes are most enriched in clusters 4 to 6, which appear to have the greatest angiogenic potential and are more closely related clusters (Fig. S16, *A*–*C*). Nonetheless, there is overlapping expression of *GATA2-AS1* with tip and stalk cell genes across clusters. Whether *GATA2-AS1* acts in a paracrine fashion is yet to be determined, although we note expression of *GATA2-AS1* in blood exosomes (Fig. 8*A*).

### *GATA2-AS1* is differentially expressed in human disease samples

Given that SNVs in *GATA2-AS1* reduce *GATA2-AS1* expression and are associated with early-onset CAD, we assessed *GATA2-AS1* expression in human disease samples. First, we assessed whether *GATA2-AS1* could function as a biomarker for CAD. We queried exoRBase (59) and found that exosomal expression of *GATA2-AS1* was higher in coronary heart disease compared with healthy populations (Healthy, WB) (Fig. 8*A* and S17*A*). Superficially, these results do not fit the paradigm that alleles with lower expression of *GATA2-AS1* in HUVEC are associated with early-onset CAD. However, it is not known how *GATA2-AS1* contributes to early-onset CAD. The molecular pathology of disease pathogenesis in early-onset CAD may be different from that found in established disease. Furthermore, the cellular source of *GATA2-AS1* is unknown in these disease samples. Finally, the risk of CAD in humans is higher (60) than the minor allele frequency of *GATA2-AS1* variants. Thus, while SNVs within *GATA2-AS1* are associated with early-onset CAD, the genetic background of samples from exoRBase may represent alternative pathogenesis of disease.

Next, we evaluated *GATA2-AS1* expression in ECs from atherosclerosis tissue from scRNAseq samples of diseased human carotid arteries (GSE159677). Compared with atherosclerotic core, there was greater expression of *GATA2-AS1* in two EC clusters from control tissue (E.3, E.4) and decreased expression in one EC cluster (E.5) (Fig. 8*B*). A similar pattern was seen with *GATA2* in clusters E.3 and E.4 but not E.5. As with our data, there was some overlap of tip/stalk cell genes in clusters that express *GATA2-AS1*, but the pattern of expression between control and atherosclerosis core was different (Fig. S17*B*). Similarly, there is differential expression of *GATA2-AS1* and *GATA2* in endothelial cells from human aortic aneurysms (Fig. S17*C*, GSE155468). Thus, the pattern of *GATA2-AS1* expression within clusters of ECs is perturbed in ECs from atherosclerotic tissue.

## Discussion

A growing body of evidence implicates lncRNAs as important mediators of endothelial biology (6, 13, 14, 15, 16, 61, 62). Here, we describe *GATA2-AS1*, an EC-enriched lncRNA that orchestrates HIF1-α-dependent and HIF1-α-independent signaling with hypoxia. One of the more abundant EC-enriched lncRNAs, *GATA2-AS1*, sets the stage for coordinated sprouting angiogenesis in hypoxia by regulating the endothelial metabolic apparatus and gene expression networks.

### Contribution of *GATA2-AS1* to the HIF switch

In most cells, HIF1-α is central to the transcriptional response to hypoxia, while in ECs, both HIF1-α and HIF2-α are required. A rapid reduction in HIF1-α leads to the switch between HIF1-α and HIF2-α signaling from acute to chronic hypoxia (43, 46). HIF1-α and HIF2-α have distinct, cell-specific responses. HIF1-α functions to maintain cell survival through its roles in metabolism and vessel sprouting, whereas HIF2-α functions to maintain tissue survival through its roles in the vascular response to hypoxia (63). Indeed, cell death ensues when HIF1-α levels are not reduced in chronic hypoxia (64). We find that *GATA2-AS1* contributes to HIF1-α, but not HIF2-α, induction with acute hypoxia. Subsequently, both *GATA2-AS1* and HIF1-α levels are reduced in chronic hypoxia. In this way, *GATA2-AS1* contributes to the balance of HIF1-α to HIF2-α in the “HIF switch” mechanism (65, 66). Accordingly, we find that *GATA2-AS1* is a key factor in the upregulation of the anerobic glycolytic pathway with hypoxia (67).

However, *GATA2-AS1* has HIF1-α-independent regulation. Even in normoxia, ECs are highly glycolytic (51) and have low mitochondrial mass, consistent with a role for endothelial mitochondria in signaling rather than energy production (52, 68). In some cell types, HIF1-α is a key factor in reducing mitochondrial mass in hypoxia through mitophagy (50). While the effects of *GATA2-AS1* on BNIP3/BNIP3L were in keeping with HIF1-α induction, *GATA2-AS1* maintains mitochondrial mass and membrane potential with hypoxia. In addition to increases in VEGF (69), these findings provide another pathway by which mitochondrial biogenesis in hypoxia is maintained in ECs (49). Thus, *GATA2-AS1* positions ECs for an appropriate metabolic response to hypoxia. HIF1-α is ubiquitously expressed across cell types (44), yet these mitochondrial dynamics are a cell-specific response. Together, these results support *GATA2-AS1* as a mediator of the EC-specific response to hypoxia.

### *GATA2-AS1* mediates a balance of angiogenic processes in hypoxia

In sprouting angiogenesis, sprouts are led by a tip cell and elongated by proliferating stalk cells (70). Both glycolysis and reactive oxygen species are metabolic drivers of the tip cell phenotype (51, 68, 70). Tip cell formation is a dynamic and competitive process (71), whereby tip and stalk selection dynamically respond to external signals on a heterogeneous background of tip and stalk susceptibility. A failure to regulate this process can lead to pathologic vascular hypersprouting (70).

*GATA2-AS1* augments HIF1-α induction and upregulates the glycolytic pathway. As glycolysis drives sprout numbers, tip cells experience a high glycolytic flux during angiogenesis. In addition, compartmentalized glycolysis within filopodia and lamellipodia facilitates migration (51). Accordingly, we found that *GATA2-AS1* increased EC migration in hypoxia. Thus, *GATA2-AS*1 may facilitate the cooperation of heterogenous ECs during angiogenesis, each following a different metabolic program (72).

These observations suggest that *GATA2-AS1* would increase tip susceptibility, sprout numbers, and pathologic hypersprouting. However, we found that *GATA2-AS1* inhibited sprout number in hypoxia. Indeed, *GATA2-AS1* inhibited the expression of both tip and stalk cell genes in keeping with scRNAseq data that *GATA2-AS1* is expressed across all EC clusters, including tip and stalk clusters. One explanation is that *GATA2-AS1*-enriched ECs are positioned to limit sprout number in an HIF1-α-independent manner. Overall, this work suggests that heterogeneous expression of *GATA2-AS1* provides one path to control the metabolic and molecular coordination of sprouting.

### Disease associations of *GATA2-AS1*

Genome-wide association studies and updated lncRNA catalogues suggest that 40.7% of lncRNAs are associated with a trait (8). Genetic variation of the lncRNA *ANRIL* (*CDKN2B-AS1*) first highlighted the potential role of lncRNAs in cardiovascular disease (73). Over a decade since this discovery, we are still learning the pathogenic contribution of *ANRIL* to this multifactorial disease (74). Here, we find a disease association for *GATA2-AS1* both with the development of cardiovascular disease and in established cardiovascular disease.

First, we find that SNVs in *GATA2-AS1* exons are associated with early-onset CAD (32) and decreased *GATA2-AS1* expression. Next, we find that *GATA2-AS1* expression is perturbed in tissue from patients with established cardiovascular disease. Depending on the cellular source, exosomes can contribute to both prevention of atherosclerosis or activation of thrombogenesis (75). Whether *GATA2-AS1* in this context plays a protective or pathogenic role remains to be determined. The data presented here represent initial studies to describe a role for the endothelial-enriched lncRNA *GATA2-AS1* in endothelial gene regulation and disease relevance. Future work with diseased tissue, disease models, and endothelial cell types will help to further elucidate the role of *GATA2-AS1* in cardiovascular disease. Overall, this evidence links *GATA2-AS1* with cardiovascular disease and provides rationale for future studies.

Overall, these findings provide further evidence for a coordinating role of lncRNAs in mediating cell type–specific responses. Future studies will be important to determine the mechanisms by which *GATA2-AS1* may contribute to disease development and/or recovery.

## Experimental procedures

### Cell culture

HUVECs at early passage (passages 2–5) were isolated and cultured from multiple independent donors as per (76) (St Michael’s Hospital, REB# 03-201). Primary cultures of endothelial cells were plated on 100-mm tissue culture dishes precoated with 0.2% gelatin. Cells were maintained in Endothelial Cell Growth Medium 2 (PromoCell, Cat# C-22011). All cell cultures were maintained at 37 °C in a humidified 5% CO_2_ chamber. For hypoxia treatment, cells were subjected to <1% O_2_ in a temperature- and humidity-controlled incubator within a sealed anaerobic system (Thermo Forma model 1025) using a high-purity anaerobic gas mixture (5% CO_2_, 10% H_2_, 85% N_2_; Linde).

Differentiation studies with human pluripotent stem cell populations were carried out in collaboration with Dr Gordon Keller’s lab in accordance with established REB approvals. Undifferentiated hPSCs included the previously described HES2-tdRFP (karyotype: 46, XX) hESC line grown under established culture conditions (41). To generate progenitor populations of arterial and venous endothelial lineages, initiation of hESC differentiation was performed in embryoid bodies following established hematovascular mesoderm patterning followed by arterial and venous specification similar to previous studies (42). Briefly, under hypoxic conditions (5% CO2, 5%O2, 90% N2) day 4 mesoderm cell populations (>60% KDR+CD56+) were treated with either artery promoting (100 ng/ml VEGFA, 30 ng/ml bFGF) or vein promoting (10 ng/ml VEGFA, 10 μM GSI [NOTCH signaling inhibitor], 30 ng/ml bFGF) optimized conditions in “base media” consisting of StemPro34 (25%v/v, Thermo Fisher), IMDM (75%v/v, Thermo Fisher), ITS-X (1:10,000, Thermo Fisher), penicillin/streptomycin (1%, Thermo Fisher), L-Glutamine (2 mM, Thermo Fisher), Ascorbic Acid (50 μg/ml, Sigma), Transferrin (150 μg/ml, Roche), and monothioglycerol (50 μg/ml, Sigma). After 8 days of culturing, the formation of CD34+CD31/PECAM1low endothelial progenitors was evident. Purification was performed with CD34-targeted magnetic activated cell sorting (Miltenyi). Cryopreserved purified angioblasts (>95% CD34+) specified to arterial (CD34+CD31/PECAM1low CD184/CXCR4+ CD73/NT5Elow) or venous (CD34+CD31/PECAM1low CD184/CXCR4- CD73/NT5E high) lineages were cultured on Matrigel-coated tissue culture plates to mediate conversion of angioblasts to ECs and expand arterial and venous endothelial cells for subsequent experiments.

ECs were cultured in base media supplemented with arterial (100 ng/ml VEGFA, 30 ng/ml bFGF) or venous (10 ng/ml VEGFA, 30 ng/ml bFGF) growth and maintenance factors. Cells were grown for 6 days (day 14 of differentiation) and at this point were dissociated and examined for endothelial purity (>80% CD34+CD31/PECAM1+) and maintenance of arteriovenous identity (arterial ECs were CD184/CXCR4 high CD73/NT5E mid, and venous ECs were CD184/CXCR4 low/negative CD73/NT5E high). Arterial-like and venous-like ECs from exclusively hypoxic culture up to this point were replated on fibronectin-coated glass slides. Cells were grown to confluence at 20% O2 for 24 h and were then moved to respective flow ± hypoxia *in vitro* setups for further study.

### RNA isolation

Total cellular RNA was harvested using the RNeasy Mini Kit (Qiagen, Cat# 74104) according to the manufacturer’s instructions. Reverse transcription was done with 100 ng to 1 μg of total RNA using the SuperScript III First-Strand Synthesis SuperMix for RT-qPCR (Thermo Fisher Scientific, Cat# 11752-050) according to the kit instructions. RT-qPCR was performed using the QuantStudio 7 Flex Real-Time PCR System (Applied Biosystems).

### Identification and validation of lncRNAs

The Human lncRNA Microarray V2.0 on Agilent 8 x 60K array platform (33,045 lncRNA probes and 30,215 protein-coding mRNA probes, Arraystar) was used to identify *GATA2-AS1* targets.

### DNA isolation, PCR, and sanger sequencing to detect single nucleotide variants

Cells were lysed using standard phenol/chloroform and salt–ethanol precipitation methods.

The primer pairs that were used to PCR amplify heterozygous SNVs from HUVEC cells using Platinum *Taq* DNA Polymerase High Fidelity (Thermo Fisher Scientific, Cat# 11304011) can be found in Table S1. PCR products were Sanger sequenced at The Centre of Applied Genomics (Peter Gilgan Centre for Research and Learning) using the corresponding primers in Table S1.

### Single molecule RNA fluorescence *In Situ* hybridization

FISH was performed using QuantiGene ViewRNA ISH Cell Assay Kit (Affymetrix, Cat# QVC0001) following the manufacturer’s instructions. HUVECs were grown to confluence on gelatin-coated coverslips, then fixed in 4% paraformaldehyde and permeabilized. Probes were applied (probes, *GATA2*: VA1-1453, *GATA2-AS1*: VA4-6001024) and nuclei were stained with DAPI. Images were collected on the Spinning Quorum Disc Confocal Microscope using an EM-CCD camera (Hamamatsu ImageEMX2) with either a 20× objective (0.6 NA) or a 63× oil immersion objective (1.4 NA). Image analysis was conducted using Imaris Image Analysis software and FIJI.

### Immunoblotting

Total cellular protein was isolated using RIPA buffer (Cell Signaling Technology). Proteins were size fractionated on NuPAGE Novex 4 to 12% Bis-Tris (Invitrogen) using Xcell SureLock Mini-Cell (Invitrogen) and transferred onto 0.45-μm nitrocellulose membranes using the Xcell II Blot Module (Invitrogen) according to manufacturer’s recommendations. Membranes were incubated overnight with the one antibodies for proteins of interest (Table S2). Horseradish peroxidase–conjugated secondary antibodies for rabbit anti-mouse IgG (ab6728, Abcam), goat anti-rabbit IgG (sc-2004, Santa Cruz Biotechnology), or donkey anti-goat IgG (sc-2020, Santa Cruz Biotechnology) were incubated for 1 h at room temperature. Signal quantification was performed using either ImageJ (NIH) or Image Lab (Bio-Rad).

### siRNA knockdown

HUVECs were grown to 90% confluency on 60-mm (2) gelatin-coated tissue culture plates and transfected with siRNA at a final concentration of 40 nM using Oligofectamine (Invitrogen) in a total volume of 2000 μl. Transfection occurred for 4 h at 37 °C in Opti-MEM medium (Invitrogen), after which M199 medium (Invitrogen) containing fetal bovine serum (Hyclone), heparin, and endothelial cell growth supplement (Biomedical Technologies) was added. Cells were incubated with siRNA for 24 to 48 h depending on the downstream application. Custom Stealth siRNAs (Invitrogen) were used to knockdown *GATA2-AS1*. SiGENOME Non-Targeting siRNA #3 (Dharmacon) was used as control siRNA transfection (Table S3).

### Single-cell RNA sequencing preparation and analysis

An independent line of passage 3 HUVECs were prepared for sequencing. Samples were prepared and submitted on the same day to Princess Margaret Genomics Centre. The Chromium Single Cell 3′ Reagent Kits v2 User Guide (CG00052) was used, and sequencing libraries targeting ∼6000 cells per sample were used. This is a droplet-cell capture approach and uses a 3′ end counting sequencing approach. Libraries were sequenced on Illumina HiSeq2500 instrument targeting 100,000 reads per cell. Raw Illumina sequencing data from Chromium Single Cell libraries were preprocessed using the CELLRANGER (v2.1.0) pipeline from 10× Genomics. FASTQ sequences were mapped to the GRCh38 human reference genome using STAR aligner (STAR v2.5.2b) (77). The quality was assessed using RNA-SeQC (v1.1.7) and SAMTOOLS (v1.3.1). Gene-barcode matrices were normalized according to the method by Lun *et al.* (78) using the R package SCRAN (v1.2.2). Low-quality cells with log-library sizes >4 median absolute deviations below the median log-library size, log-number of genes detected >4 median absolute deviations below the median, or high mitochondrial gene expression (UMI counts) were removed from the data sets. A total of 8281 cells passed filtering. Approximately 3627 genes were measured per cell with 37,151 reads per cell. Principal component analysis, clustering, differential gene expression, and universal manifold approximation and projection visualizations were performed using R packages, SCATER (v1.2.0) (79), CELLRANGERRKIT (v1.1.0) (80), RTSNE (v0.11), SC3 (v1.3.14) (81), EDGER (v3.16.5) (82), SEURAT (v2.1) (83) and PCAMETHODS (v1.50.0) (83). Loupe Cell Browser (v2.0.0) was used to view and explore the dataset to find significant genes, cell types, and subpopulations. Data for carotid atherosclerosis samples were accessed on GEO (GSE159677). Data for aortic aneurysm samples were accessed on GEO (GSE155468).

### RNA secondary structure prediction

RNA secondary structure predictions were made using RNAstructure (33) (http://rna.urmc.rochester.edu/RNAstructureWeb/Servers/Predict1/Predict1.html accessed September 15, 2019).

### Poly-adenylated RNA fractionation

PolyA RNA was isolated using the PolyA Spin mRNA Isolation Kit (New England Biolabs). RNA samples were purified as described above for five biological replicates of HUVEC. RNA was fractionated according to manufacturer’s instructions. cDNA was made from equal proportions of RNA based on the total mass collected from each fraction.

### Nuclear/cytoplasmic partitioning

HUVECs were detached with 0.05% Trypsin and washed with PBS. A volume of 175 μl RLN buffer (50 mM Tris-HCl, pH 8.0; 140 mM NaCl; 1.5 mM MgCl2; 0.5% Nonidet P-40; 0.2 units/μl RNaseOUT; 1 mM dithiothreitol [DTT]) was used to resuspend pellet followed by a 5-min incubation on ice. Cellular debris and nuclei were pelleted at 300*g* for 2 min at 4 °C. The cytoplasmic fraction was transferred to 600 μl Solution D (4 M guanidinium thiocyanate, 25 mM sodium citrate, pH 7; 0.5% sarcosyl, 0.1 M 2-mercaptoethanol) (84). The nuclear pellet was washed in 500 μl PBS, pelleted again at 300*g* for 3 min at 4 °C, and resuspended in 600 μl Solution D. Both nuclear and cytoplasmic fractions were then subjected to RNA isolation as described above. Quantification of nuclear and cytoplasmic fractions was normalized relative to an equal percentage of RNA extracted from each fraction.

### Transwell migration assay

Twenty-four Transwell plates (8 um pore size; BD Biosciences) were coated with 0.2% gelatin, and 10% fetal bovine serum was used as a chemoattractant in the lower chamber to initiate cell migration. A total of 50,000 HUVECs were plated and exposed to either normoxia or hypoxia for 16 h. The top of the inserts were cleared of cells with a cotton swab, fixed with 10% v/v of Formalin in PBS for 5 min. Migrated cells on the bottom of the insert were stained with 0.2% Crystal violet for 5 min. The migrated cells were quantified by counting six representative fields of each insert using scanned images (20× magnification). Results are expressed as an average of cell count per field, performed using two biological replicates with n = 9 inserts.

### Nanostring

A Nanostring gene expression array was used to study the mRNA expression of 272 genes important in endothelial biology. A custom nCounter XT CodeSet was designed by the Bioinformatics team at Nanostring Technologies targeting 272 genes important in endothelial biology. RNA integrity number of the samples was assessed using the Agilent 2100 Bioanalyzer System. All samples had an RNA integrity number of 7.5 or higher, ensuring that intact RNA was used for the assay. The nCounter XT assay was performed as per the manufacturer’s instructions. Data analysis was done using the nSolver Analysis Software provided by Nanostring. Quality control examined imaging efficiency, binding density, hybridization efficiency, and background fluorescence. Only samples that passed all quality control checkpoints were included in the analysis. Transcript counts were corrected for background fluorescence and normalized to synthetic positive controls to reduce protocol-related variations. Transcript counts were normalized to four housekeeping genes (*B2M*, *HPRT*, *SDHA*, and *TBP* that had a coefficient of variability <30%.

### Mitochondrial assays

Mitotracker Green FM, 20 nM (Thermo Fisher, M7514) was used to stain HUVECs according to manufacturer’s recommendations. JC-1, 5 μg/ml (Cayman Chemical, 15003) was used to stain HUVECs as in (85). Briefly, HUVECs were treated with siRNA for 24 h A total of 20,000 HUVECs were split into black-walled, clear bottom 96-well plates and left to adhere for 1 h. Cells were then treated with normoxia or hypoxia for a total of 24 h. Cells were washed with PBS+/+ and stained with either Mitotracker Green FM or JC-1 for 30 min and washed again with PBS+/+. Fluorescence was measured with a platereader (BioTek Multi-Mode HTX).

### Spheroid assay

HUVECs were transfected with siRNA for 24 h and then trypsinized, resuspended in HUVEC medium, and counted with the Vi-Cell XR Cell Analyzer (Beckman Coulter). Using approximately 750 cells per spheroid, the cells were combined with M199 and methylcellulose [(Cells + M199 = 80%) + (methylcellulose = 20%)]. Spheroids were generated *via* the “hanging drop” method in an incubator at 37 °C overnight. Spheroids were then pooled together and centrifuged at 500*g* for 3 min in PBS + 10% fetal bovine serum. The spheroids were then combined with methylcellulose, type I collagen (pH = 7), and VEGFA (100 ng/ml). The mixture was next plated onto an eight-well u-slide (Ibidi) and incubated at 37 °C for 6 h, then they either remained in normoxia (21% O_2_) or were transferred to hypoxia (1% O2) for 18 h. Spheroids were imaged using the Zeiss AxioObserver Live Cell with FLIM or the Nikon Eclipse TS100 with a 20× objective. Images were processed using FIJI.

### ExoRBase

*GATA2-AS1* and GATA2 were searched on exoRBase (http://www.exorbase.org accessed June 23, 2021) (59). Visualizations of individual-level expression of *GATA2-AS1* and *GATA2* were exported as PDF. Individual level data were also exported, and differential expression between groups was plotted in GraphPad 8. Statistical difference between groups was calculated with one-way ANOVA in GraphPad 8.

### Statistics

Unless otherwise stated, all experiments were performed in a minimum of three biological replicates. Unless otherwise indicated, the mean was calculated, and error bars indicate standard error (SEM). A *p*-value <0.05 was considered statistically significant.

## Data availability

Microarray data are available under GEO Accession: GSE197376.

## Supporting information

This article contains supporting information (27, 41, 42).

## Conflict of interest

The authors declare that they have no conflicts of interest with the contents of this article.
