# Supplementary material for: Long noncoding RNA GATA2-AS1 augments endothelial hypoxia inducible factor 1-α induction and regulates hypoxic signaling
Source: J Biol Chem. 2023 Feb 17;299(5):103029. doi: 10.1016/j.jbc.2023.103029 (PMC10148162; doi:10.1016/j.jbc.2023.103029)
Supplement: Supporting Table S1 [file mmc3.docx]

| *Gene* | Forward primer sequence | Reverse primer sequence |
| --- | --- | --- |
| *Firefly luciferase* | 5'-ACTCCTCTGGATCTACTGGTC-3' | 5'-GTAATCCTGAAGGCTCCTCA-3' |
| *Cyclophilin A* | 5'-TCTGCTTTTGGGACCTTGT-3' | 5'-GACGGCGAGCCCTTGG-3' |
| *TBP* | 5' CGCCAGCTTCGGAGAGTTC-3' | 5'- GCAATGGTCTTTAGGTCAAGT TTACA -3' |
| *VEGF-A* | 5'-GCAGACCAAAGAAAGATAGAC CAAG-3' | 5'-CGC CTC GGC TTG TCA CAT 3' |
| *GATA2-AS1 ex1/2* | 5'-TGGCGTCCGTTTGTCTGTC-3' | 5'-TCCGCGGCGATCTCATTTT-3' |
| *GATA2-AS1_ex2/3* | 5'-CGCGGCTCTCAGGGAAGTG-3' | 5'-CGGATCTGCGGGGGTAAT-3' |
| *GATA2* | 5'- GCGCAGCAAGGCTCGTTCC-3' | 5'- CCGTCCCGCCGCCAGAGAG-3' |
| *18S* | 5'- AGGAATTGACGGAAGGGCAC-3' | 5'- GGACATCTAAGGGCATCACA-3' |
| *H19* | 5'- CGTGACAAGCAGGACATGA-3' | 5'- GGAGGAAGTAAAGAAACAGAC-3' |
| *XIST* | 5'- ACGCTGCATGTGTCCTTAGTA-3' | 5'- GCAACCCATCCAAGTAGAT-3' |
| *STEEL* | 5'-GAGCCCTGCTTCCCAGATC -3' | 5'-GCCAGGTCCAGCATGAAAC -3' |
| *VEGFR2* | 5'- TTACTATTCCCAGCTACATGATCAG -3' | 5'- AGACGGACTCAGAACCACATCATAA -3' |
| *VEGFR1 (full)* | 5'- CAGTGTGAGCGGCTCCCTTATG-3' | 5'- CACAGTCCGGCACGTAGGTGAT -3' |
| *sVEGFR1* | 5'-AAGATTCAGGCACCTATG-3' | 5'-AATTTGGAGATCCGAGA – 3' |
| *DLL4* | 5'-CTGCTGCTGGTGGCACTTT-3' | 5'-CGCGCTCGTTGATGAACTC-3' |
| *CD34* | 5'- TTCAACCACTAGCACTA-3' | 5'-TTTCACTTCTCTGATGC-3' |
| *Notch4* | 5'- TGCTGCTGCTATGTGTCTCA-3' | 5'- TCCCTTGTCCCAGAGACAG-3' |
| *Notch1* | 5'-CCGTGTGGCCTCCTTCTACTG-3' | 5'-CGCAGTTGGAGCCCTCGTTACA-3' |
| *CXCR4* | 5'-CCAGTAGCCACCGCATCT-3' | 5'-ATAGTCCCCTGAGCCCATTT-3' |
| *TIE2* | 5'- CAGCTTGCTCCTTTCTGGAACT-3' | 5'- CTATGGTGATGGGCTCATGG -3' |
| *ANGPT2* | 5'-TGCCACGGTGAATAATTCAG-3' | 5'- TTCTTCTTTAGCAACAGTGGG-3' |
| *HIF-1α* | 5'-AGGCCGCTCAATTTATGAAT -3' | 5'- TTTGGCAAGCATCCTGTACT -3' |
| *HIF-2α* | 5'-TGGCGACATGATCTTTCTGTC A-3' | 5'- ATGGTCGCAGGGATGAGTGA-3' |
| *GLUT1* | 5'-CACCACCTCACTCCTGTTACTT-3' | 5'- CAAGCATTTCAAAACCATGTT TCTA-3' |
| *BNIP3* | 5'-CAGGGCTCCTGGGTAGAAC-3' | 5'-CTCCGTCCAGACTCATGCTG-3' |
| *BNIP3L* | 5'- CTGCACAAACTTGCACATTG-3' | 5'- TAATTTCCACAACGGGTTCA -3' |
| *LDHA* | 5'-GGCTGGGAGTTCACCCATTAA-3' | 5'-CTGGGTGCAGAGTCTTCAG -3' |
| *HK1* | 5'-GCGGGTGCAAGTGAATCAT-3' | 5'- AGGCACTCAGCAACATGAT-3' |
| *HK2* | 5'-GAGCCACCACTCACCCTAC-3' | 5'-TGTCCTCAGGGATGGCATAGA-3' |
| *MCT4* | 5'-AGCGACACAGCCTGGATCT-3' | 5'- GAC CCC CCA CAA GCA TGA-3' |
| *PGK1* | 5'-CTGGACAAGCTGGACGTTAAA | 5'-CTCCATTGTCCAAGCAGAATT |

**Supplemental Table 1 – Primers for RT-qPCR**
