# Supplementary material for: Long noncoding RNA GATA2-AS1 augments endothelial hypoxia inducible factor 1-α induction and regulates hypoxic signaling
Source: J Biol Chem. 2023 Feb 17;299(5):103029. doi: 10.1016/j.jbc.2023.103029 (PMC10148162; doi:10.1016/j.jbc.2023.103029)
Supplement: Supporting Table S2 [file mmc4.docx]

| Antibody | CAT. # | 1º Antibody | Dilution | Detector | Manufacturer |
| --- | --- | --- | --- | --- | --- |
| GATA2 | SC9008 | Rabbit Polyclonal | 1:200 | HRP | Santa Cruz |
| HIF1α | AF1935 | Goat Polyclonal | 1:400 | HRP | R&D Biosystems |
| HIF2α | NB100-122 | Rabbit Polyclonal | 1:500 | HRP | Novus |
| ß-actin | 4970 | Mouse monoclonal | 1:1000 | HRP | Cell Signaling |
| α-tubulin | Ab11304 | Mouse monoclonal | 1:1000 | HRP | Abcam |
| HKII | 2106 | Rabbit polyclonal | 1:1000 | HRP | Cell Signaling |
| LDHA | 2012 | Rabbit polyclonal | 1:1000 | HRP | Cell Signaling |
| BNIP3L | Ab8399 | Rabbit polyclonal | 1:1000 | HRP | Abcam |
| BNIP3 | Ab10433 | Mouse monoclonal | 1:600 | HRP | Abcam |

**Supplemental Table 2 – Antibodies for Western Blotting**
