# Supplementary material for: Long noncoding RNA GATA2-AS1 augments endothelial hypoxia inducible factor 1-α induction and regulates hypoxic signaling
Source: J Biol Chem. 2023 Feb 17;299(5):103029. doi: 10.1016/j.jbc.2023.103029 (PMC10148162; doi:10.1016/j.jbc.2023.103029)
Supplement: Supporting Table S3 [file mmc5.docx]

| **siRNA** | **Sequence** |
| --- | --- |
| **GATA2-AS1 siRNA A** | 5’ – CAGCGCAGCUUACGAUUCUUCAUCA – 3’ |
| **GATA2-AS1 siRNA B** | 5’ – UUUCAGAGGGUCUUGCUAGUCUCCG – 3’ |
| **GATA2-AS1 siRNA C** | 5’ – CCCUUUCAGAGAGCGCACAACAAAU – 3’ |
| **GATA2-AS1 siRNA D** | 5’ – CGCCCGCCAUCGAAUGCAUCAUUUA – 3’ |
| **GATA2 siRNA A** | 5’ – GCAAGGCUCGUUCCUGUUC – 3’ |
| **GATA2 siRNA B** | 5’ – GAAGGGAUCCAGACUCGGA – 3’ |
| **GATA2 siRNA C** | 5’ – GCACAAUGUUAACAGGCCA – 3’ |
| **GATA2 siRNA D** | 5’ – GGGCAGAACCGACCACUCA – 3’ |

**Supplemental Table 3 – siRNA sequences for GATA2-AS1**
