# Supplementary material for: Long noncoding RNA GATA2-AS1 augments endothelial hypoxia inducible factor 1-α induction and regulates hypoxic signaling
Source: J Biol Chem. 2023 Feb 17;299(5):103029. doi: 10.1016/j.jbc.2023.103029 (PMC10148162; doi:10.1016/j.jbc.2023.103029)
Supplement: Supporting Table S4 [file mmc6.docx]

**Supplemental Table 4 – Examples of 1000G data**

| Individual identifier on 1000G | Subpopulation cohort | Genotype at +689 (rs66606131) | Genotype at +729  (rs6789646) | Genotype at +775 (rs6776756) | Genotype at +846  (rs6776863) | Haplotype |
| --- | --- | --- | --- | --- | --- | --- |
| HGO3012 | BEB | A/A | T/T | G/G | G/G | ATGG |
| HGO380 | FIN | A/A | T/T | G/G | G/G | ATGG |
| HG02603 | SAS | G/G | C/C | A/A | T/T | GCAT |
| HG00607 | EAS | G/G | C/T | A/A | T/T | GCAT/GTAT |
| HG003060 | AFR | A/A | T/T | G/G | G/G | ATGG |
| HG00187 | EUR | G/G | T/C | A/A | T/T | GTAT/GCAT |
| NA11881 | CEU | A/G | T/T | G/G | G/T | ATGG/GTGT |
| HG01350 | CLM | G/G | T/T | A/A | T/T | GTAT |
| HG00240 | GBR | G/G | C/C | A/A | T/T | GCAT |
| HG00244 | GBR | G/A | T/T | A/G | T/G | GTAT/ATGG |
| NA21103 | GIH | G/A | C/T | A/G | T/G | GCAT/ATGG |
| HG01672 | IBS | A/A | T/T | G/G | G/G | ATGG |
| NA18985 | JPT | A/G | T/T | G/A | G/T | ATGG/GTAT |
| HG02086 | KHV | G/G | C/T | A/A | T/T | GCAT/GTAT |
| HG01259 | AMR | G/A | C/T | A/G | T/G | GCAT/ATGG |
| NA20535 | TSI | G/G | C/C | A/A | T/T | GCAT |
| NA20813 | TSI | G/G | C/T | A/A | T/T | GCAT/GTAT |
| HGO1392 | PUR | G/A | T/T | G/G | G/G | GTGG/ATGG |
| HG02262 | PEL | G/G | T/T | A/A | T/T | GTAT |
| NA18557 | CHB | A/A | T/T | G/G | G/G | ATGG |
| HG00566 | CHS | A/G | T/C | G/A | G/T | ATGG/GCAT |
| HG04060 | SAS | A/A | T/T | G/G | G/G | ATGG |
| HG03193 | ESN | G/G | C/C | A/A | T/T | GCAT |
| NA19399 | LWK | G/A | T/T | A/G | T/G | GTAT/ATGG |
| HG02891 | MAG | A/G | T/T | G/A | G/T | ATGG/GTAT |
| HG01958 | ACB | G/G | C/T | A/A | T/T | GCAT/GTAT |
| HG02107 | ACB | G/G | C/C | A/A | T/T | GCAT |
| NA20282 | ASW | G/G | T/T | A/A | T/T | GTAT |
| HG01815 | CDX | G/G | T/T | A/A | T/T | GTAT |
| HG02164 | CDX | G/A | C/T | A/G | T/G | GCAT/ATGG |
| HG03870 | ITU | A/A | T/T | G/G | G/G | ATGG |
| HG04002 | ITU | A/G | T/C | G/A | G/T | ATGG/GCAT |
| HG03088 | MSL | G/G | T/T | A/A | T/T | GTAT |
| HG03212 | MSL | A/G | T/T | G/A | G/T | ATGG/GTAT |
| NA19681 | MXL | G/A | T/T | G/G | T/G | GTGG/ATGG |
| NA19773 | MXL | A/A | T/T | G/G | G/G | ATGG |
| HG02688 | PJL | A/A | T/T | G/G | G/G | ATGG |
| HG03238 | PJL | G/A | T/T | A/G | T/G | GTAT/ATGG |
| HG03694 | STU | A/A | T/T | G/G | G/G | ATGG |
| HG03844 | STU | G/A | T/T | A/G | T/G | GTAT/ATGG |
| NA18523 | YRI | A/G | T/T | G/A | G/T | ATGG/GTAT |
| NA19190 | YRI | G/G | C/C | A/A | T/T | GCAT |
