# Supplementary material for: Long noncoding RNA GATA2-AS1 augments endothelial hypoxia inducible factor 1-α induction and regulates hypoxic signaling
Source: J Biol Chem. 2023 Feb 17;299(5):103029. doi: 10.1016/j.jbc.2023.103029 (PMC10148162; doi:10.1016/j.jbc.2023.103029)
Supplement: Supporting Figures S1–S14 [file mmc7.pdf]

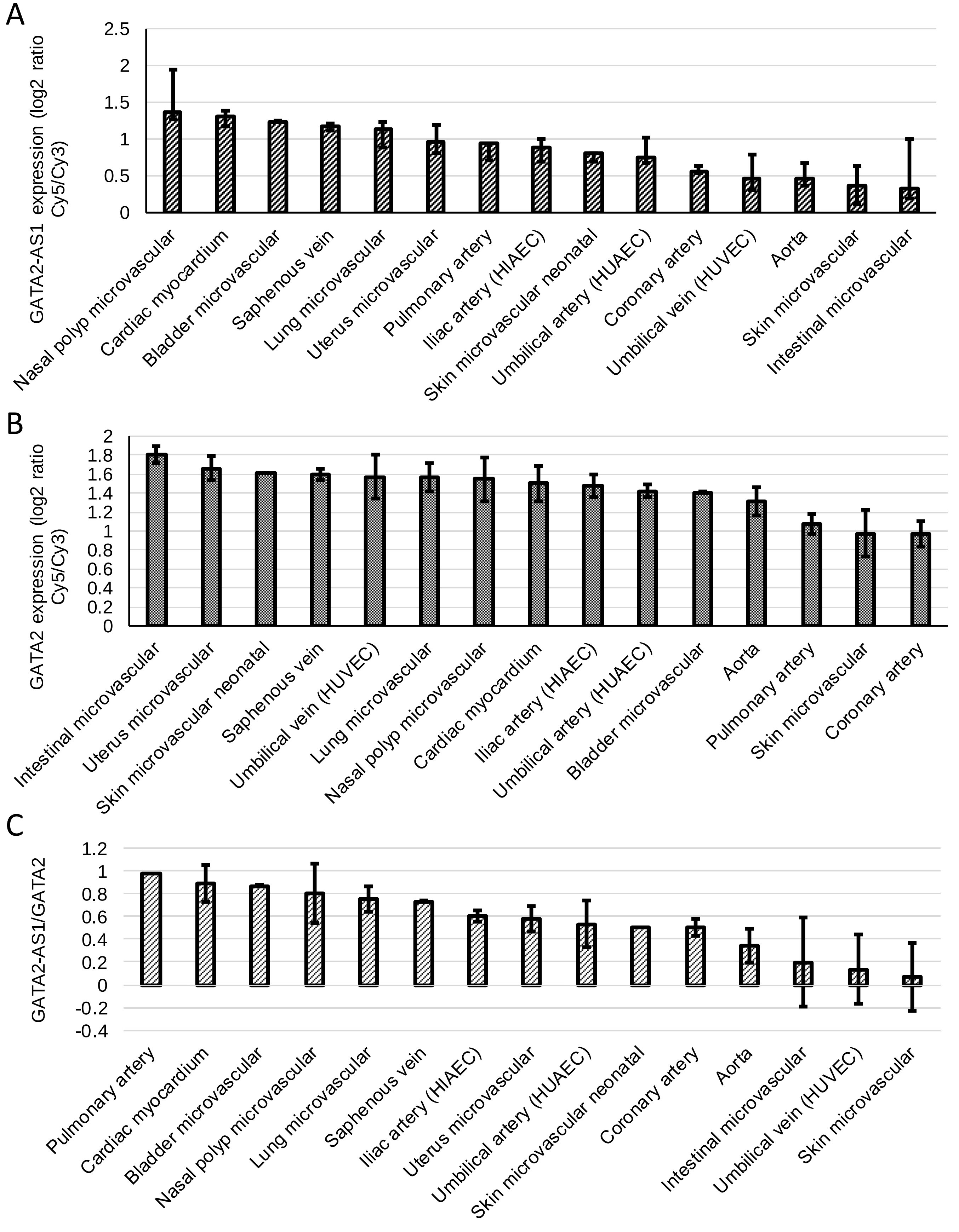

Figure S1. Expression of GATA2-AS1 and GATA2 in ECs. Microarray analysis of public data show expression of GATA2-AS1 (A) and GATA2 (B) and ratio of GATA2-AS1/GATA2 (C) in ECs derived from various vascular beds[24]. Some vascular beds show similar relative expression of GATA2-AS1 and GATA2 (saphenous vein, lung microvascular) while others show disparate relative expression (intestinal microvascular).

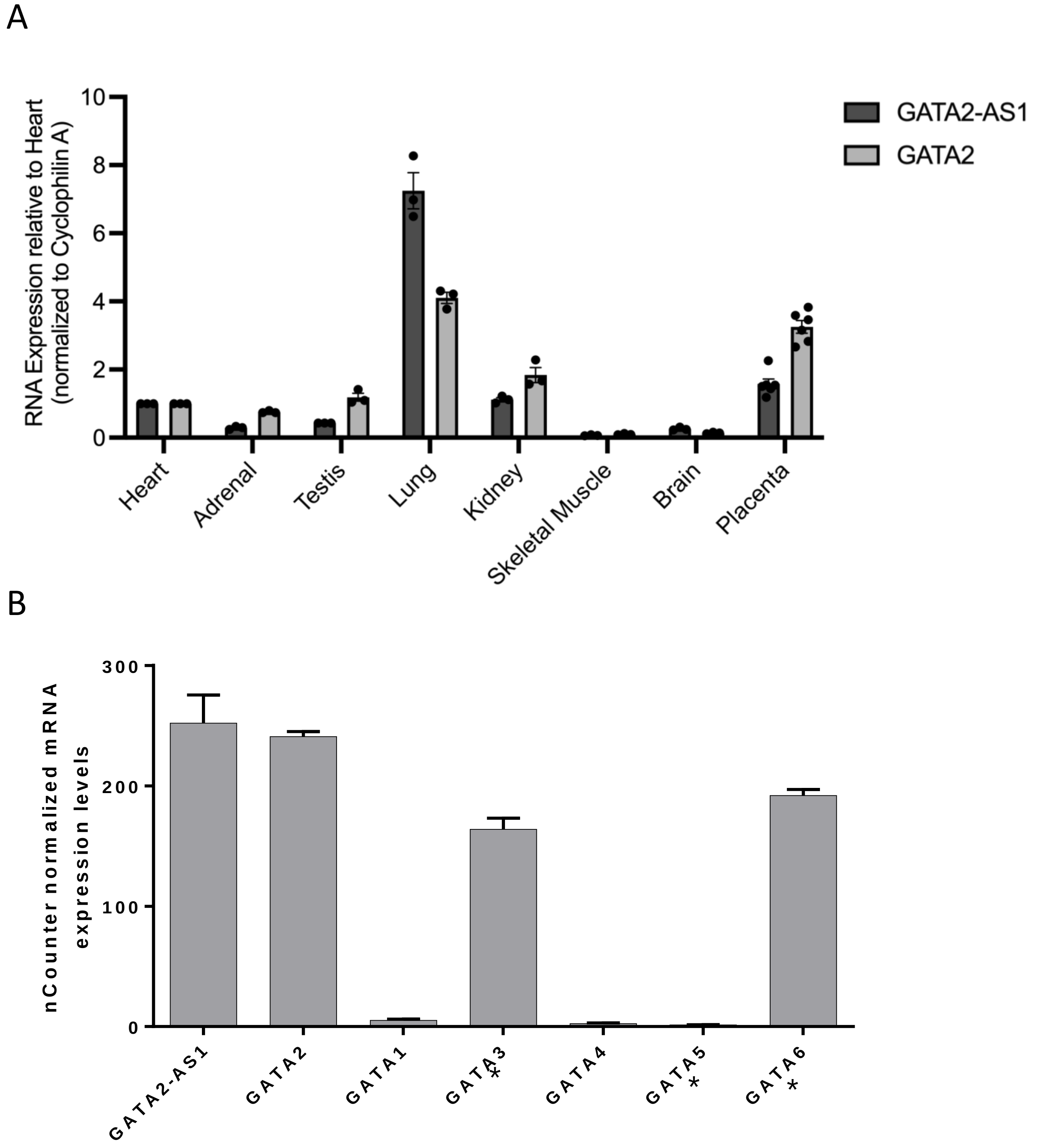

Figure S2. Expression of GATA2-AS1 across tissues and compared to GATA factors. (A) GATA2-AS1 and GATA2 are most highly expressed in the lung and have lower expression in skeletal muscle and brain. Whilst some differences in relative expression exist, GATA2-AS1 and GATA2 follow similar expression patterns across tissues. (B) Nanostring counts of GATA2-AS1 and GATA factors in HUVEC. GATA2-AS1 and GATA2 are the most highly expressed whilst GATA1, GATA4, and GATA5 are minimally expressed in HUVEC.

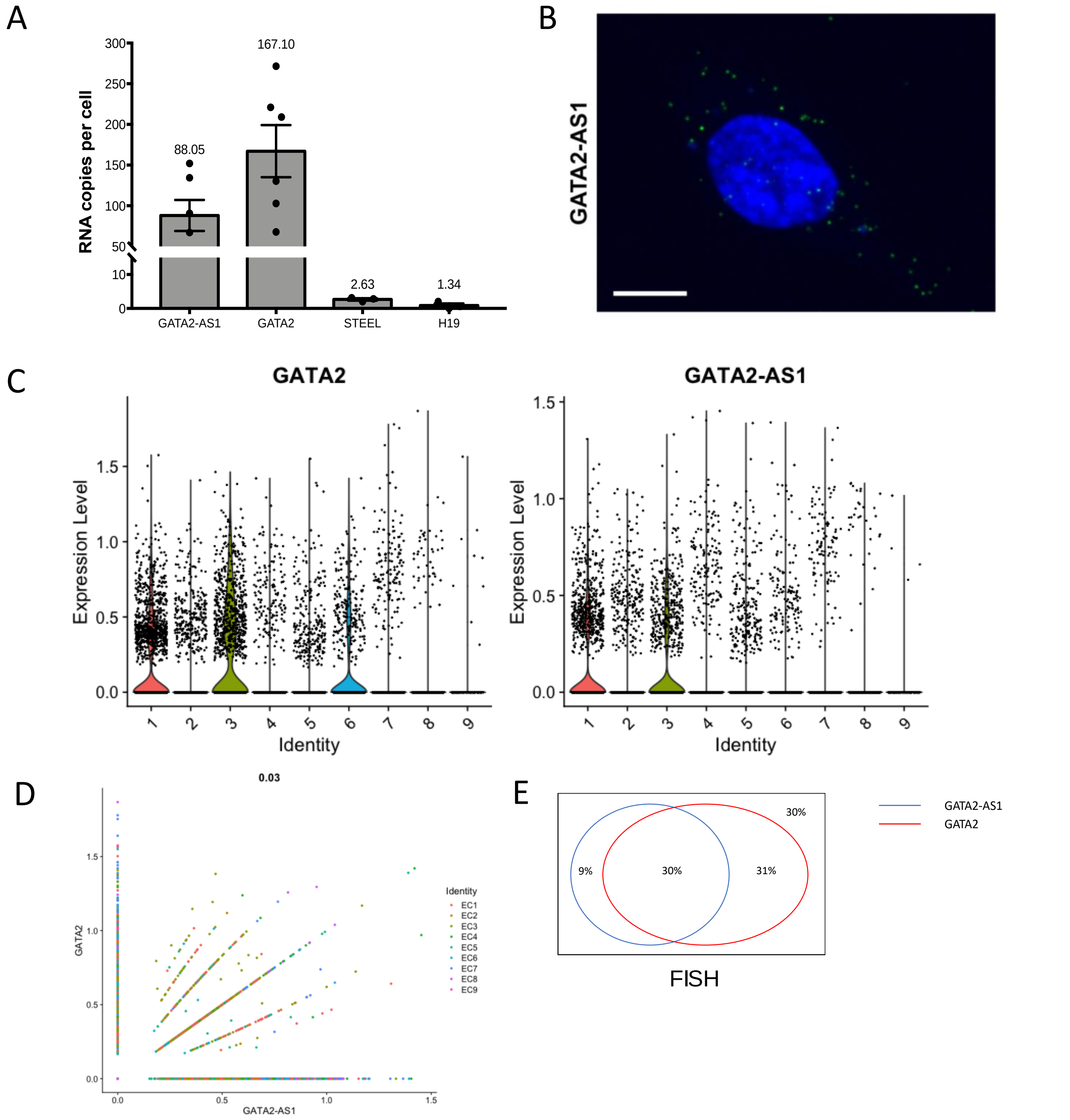

Figure S3. Expression of GATA2-AS1 and GATA2 in HUVEC. (A) Absolute copy number per cell of GATA2-AS1, GATA2, STEEL (HOXD-AS1) and H19 by RT-qPCR. GATA2-AS1 is an abundant endothelial-enriched lncRNA. Statistical significance was assessed by 2-sided Student *t* test and * $P < 0.05$. (B) Single molecule RNA fluorescent *in situ* hybridization (smRNA-FISH) of GATA2-AS1 in HUVEC imaged at 63x magnification. GATA2-AS1 (green) is primarily cytoplasmic and abundantly expressed. Green = GATA2-AS1, Blue = DAPI. Scale bar = 10μm. Images taken with Spinning Quorum Disc Confocal Microscope with a 63X oil immersion objective with 1.4 numerical aperture at room temperature and an EM-CCD camera (Hamamatsu ImageEMX2). Stains used were: DAPI (405), Green/GATA2-AS1 (Alexa488). Images were analyzed in Imaris Image Analysis Software and FIJI. (C) Violin plots from single cell RNA sequencing (scRNAseq) of HUVEC showing common expression of GATA2-AS1 and GATA2 in some clusters (Cluster 1, Cluster 3) and distinct expression of only GATA2 in other clusters (Cluster 6). (D) Correlation plot from scRNAseq of HUVEC comparing GATA2 and GATA2AS1. Pearson correlation is shown above the plot. (E) Venn diagram showing relationship between GATA2-AS1 and GATA2 expression in single cells. From smRNA-FISH, both GATA2-AS1 (expressed in ~39% of cells) and GATA2 (expressed in ~61% of cells) are expressed in a subset of HUVEC at passage 3. Most cells that express GATA2-AS1 also co-express GATA2 (~78% of cells that express GATA2-AS1). About half (~50%) of cells that express GATA2 co-express GATA2-AS1.

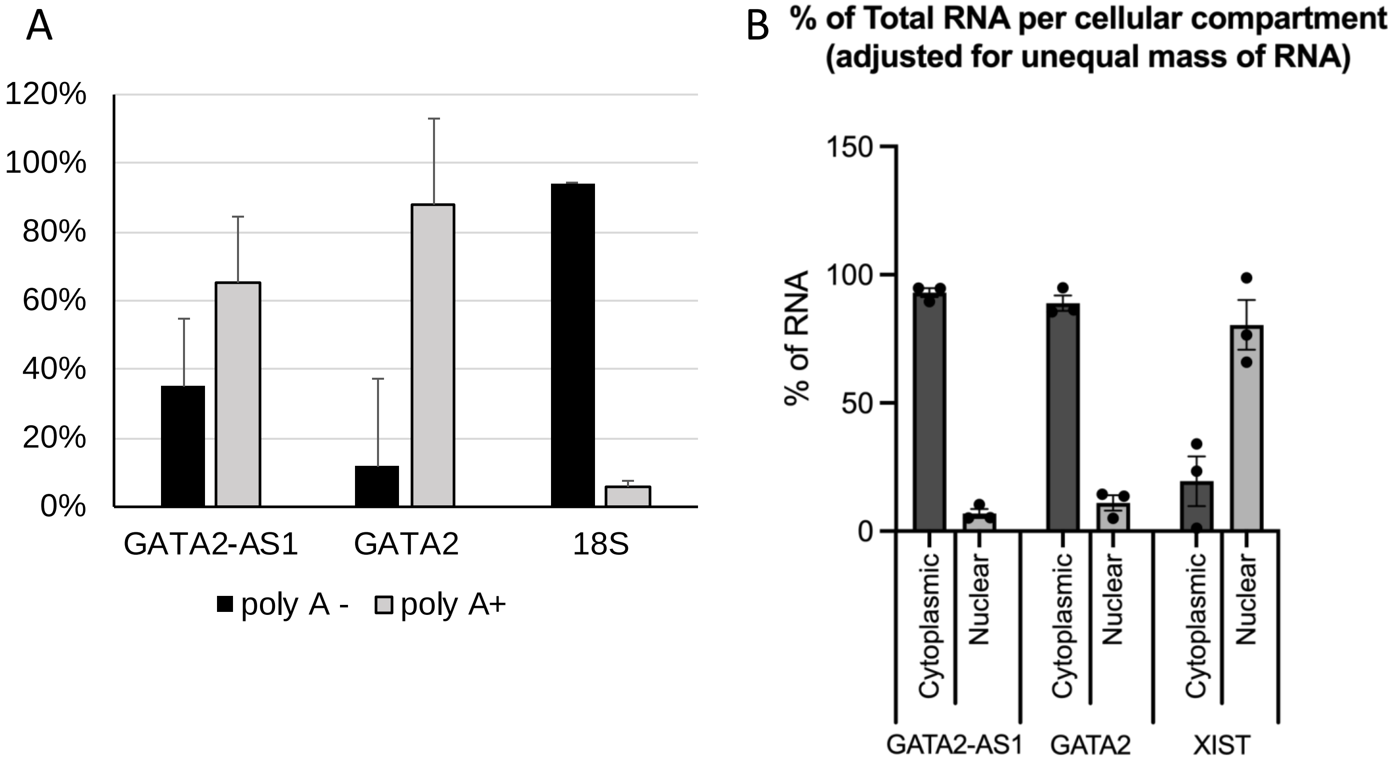

C

Gene	Gene ID	Length (nts)	Coding Potential (CPAT)		Longest in-frame ORF	Subcellular Location	BLASTX Homology Present
			Classification	Coding Probability*			
**TINCR**	NR_027064	3747	noncoding	0.204	151 AA	Cytoplasm	yes
**MALAT1**	NR_002819	8708	noncoding	0.014	92 AA	Nucleus	yes
**STEEL**	NR_110458	4078	noncoding	0.023	85 AA	Nucleus	no
**XIST**	NR_001564	19296	noncoding	0.027	136 AA	Nucleus	yes
**HOTAIR**	NR_047518	2337	noncoding	0.413	106 AA	Mixed	no
**GATA2-AS1**	NR_125398	1163	noncoding	0.268	103 AA	Cytoplasm	no
**GATA2**	NM_001145662	2525	coding	0.999	480 AA	Cytoplasm	yes
**eNOS**	NM_000603	4388	coding	1.000	1203 AA	Cytoplasm	yes

Figure S4. Bioinformatic and experimental data suggest that GATA2-AS1 is a polyadenylated, cytoplasmic long noncoding RNA. (A) RT-qPCR of GATA2-AS1, GATA2, and 18S RNA in poly A- and poly A+ RNA generated from HUVEC. GATA2-AS1 RNA is primarily found in the poly A+ fraction. GATA2 is shown as a poly A+ mRNA and 18S is shown as a poly A- RNA. (B) RT-qPCR of RNA after subcellular fractionation of HUVEC. GATA2-AS1 RNA is primarily found in the cytoplasmic fraction. GATA2 is shown as a cytoplasmic mRNA and XIST is shown as a predominantly nuclear lncRNA. (C) Bioinformatic assessment of protein-coding potential. The Coding Potential Assessment Tool (CPAT) [27] was used to assess protein-coding potential of GATA2-AS1. A threshold of 0.364 is used to separate coding and noncoding. Other lncRNAs and protein-coding genes, including GATA2, are shown for comparison. There is no BLASTX homology for GATA2-AS1. The longest in frame open reading frame (ORF) is 103 amino acids (AA) in GATA2-AS1.

A

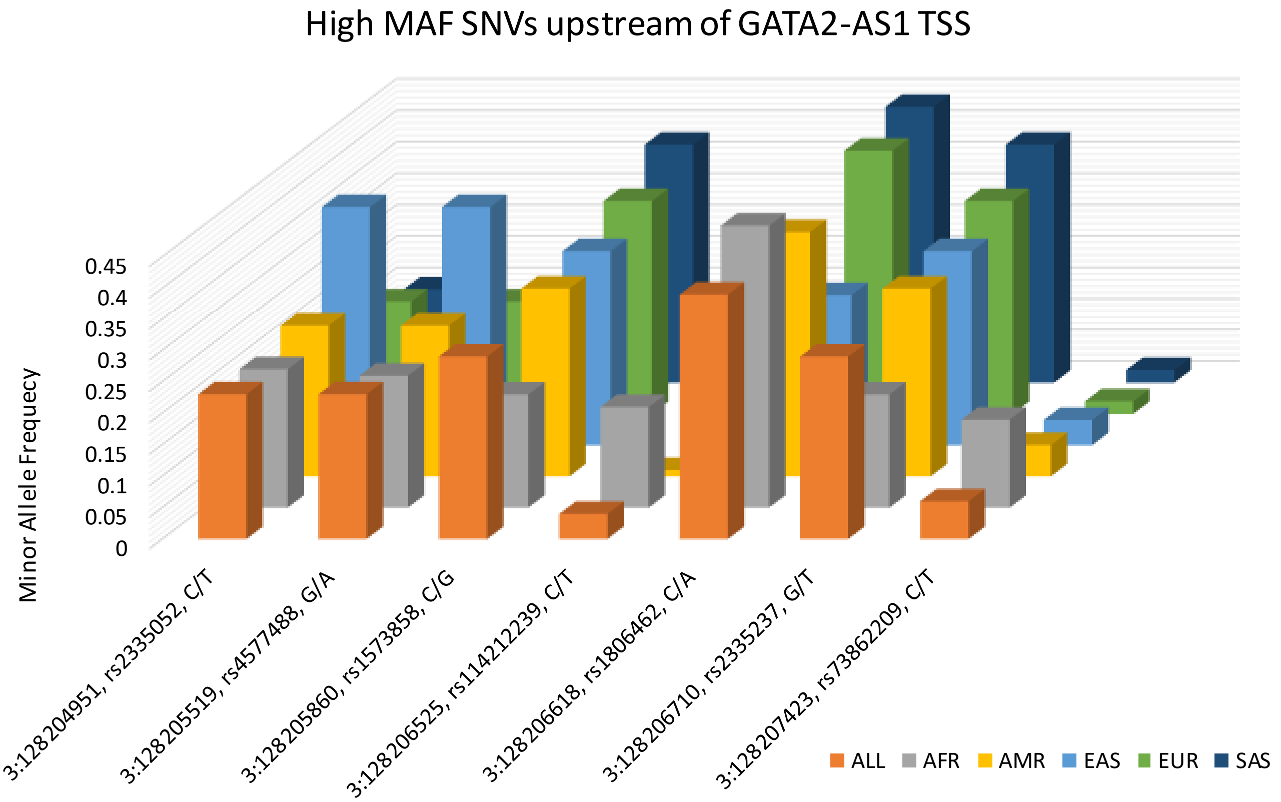

B

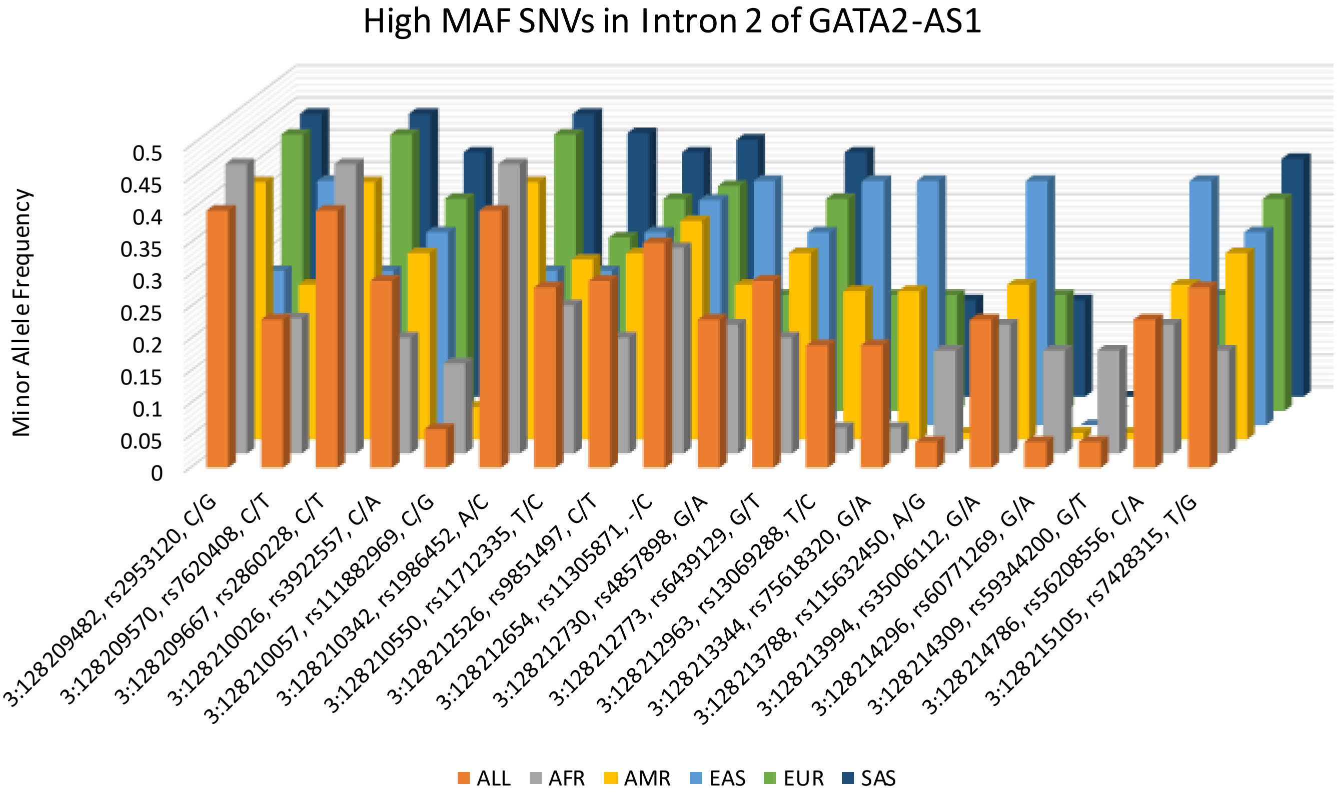

Figure S5. Variation in GATA2-AS1 upstream and intron sequences. (A) Single nucleotide variations (SNVs) with common (>5%) minor allele frequency (MAF) in GATA2-AS1 upstream sequences from the 1000 genomes project (www.1000genomes.org accessed June 20, 2019). (B) SNVs with common MAF in GATA2-AS1 intron 2. AFR = Africa, AMR = America, EAS = East Asian, EUR = European, SAS = South Asian.

A

SNP	Major allele	Minor allele	Position relative to TSS*	Minor Allele Frequencies (MAF)					
				ALL	AFR	AMR	EAS	EUR	SAS
**rs66606131**	G	A	+689	0.27	0.11	0.28	0.3	0.33	0.37
**rs6789646**	T	C	+729	0.40	0.46	0.40	0.24	0.43	0.44
**rs6776756**	A	G	+775	0.31	0.19	0.31	0.34	0.37	0.38
**rs6776863**	T	G	+846	0.28	0.16	0.29	0.30	0.33	0.37

B

Minor haplotypes found in 1000G cohort

	rs66606131	rs6789646	rs6776756	rs6776863	
**Haplotype**	+689 G/A	+729 T/C	+775 A/G	+846 T/G	Haplotype Frequency (%)
**4**	G	T	G	T	2.9
**5**	G	T	G	G	1.2
**6**	A	C	G	G	0.1

C

Major haplotypes found in HUVEC samples

	rs66606131	rs6789646	rs6776756	rs6776863	
**Position relative to TSS**	+689	+729	+775	+846	Haplotype Frequency (%)
**Major Allele/Minor Allele**	G/A	T/C	A/G	T/G	
**1**	G	T	A	T	31.25
**2**	G	C	A	T	43.75
**3**	A	T	G	G	25

D Haplotypes 1/2: GTAT/GCAT

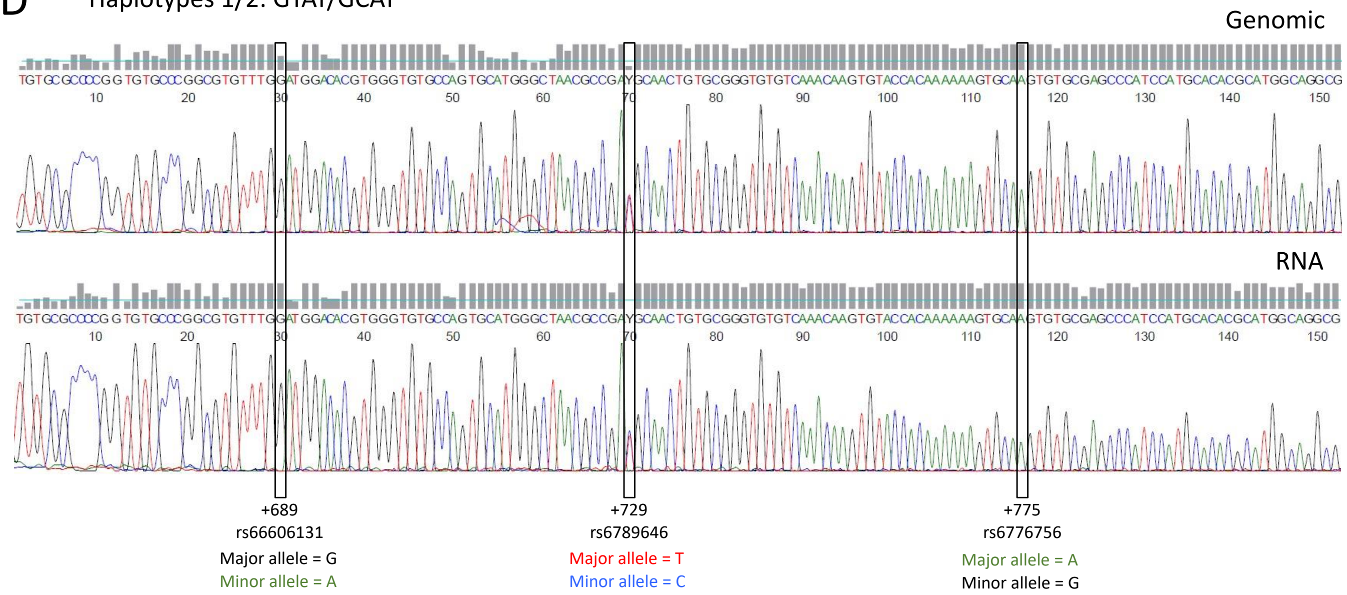

Figure S6. Genetic variation in GATA2-AS1 and expression of GATA2-AS1. (A) Four SNVs within GATA2-AS1 exons have MAFs > 5% across continents. (B) Frequency of three minor haplotypes of SNVs within GATA2-AS1 across the 1000 genomes dataset (n = 2506). (C) Frequency of common haplotypes of SNVs within GATA2-AS1 across sequenced primary HUVEC (n=8). Three major haplotypes are seen. Four SNVs within GATA2-AS1 exons are indicated with their positions vs the transcription start site (+689, +729, +775, +846). (D) Sequencing of RNA (cDNA) and genomic DNA from HUVEC. Examples of a heterozygote for haplotype 1/2 is shown. The two haplotypes include haplotype 1 with the major allele at all four SNV sites (GTAT) or haplotype 2 with the minor allele at position +729 only (GCAT). Both haplotypes in this example show equal heights for sequencing peaks of RNA at variant positions indicating equal expression.

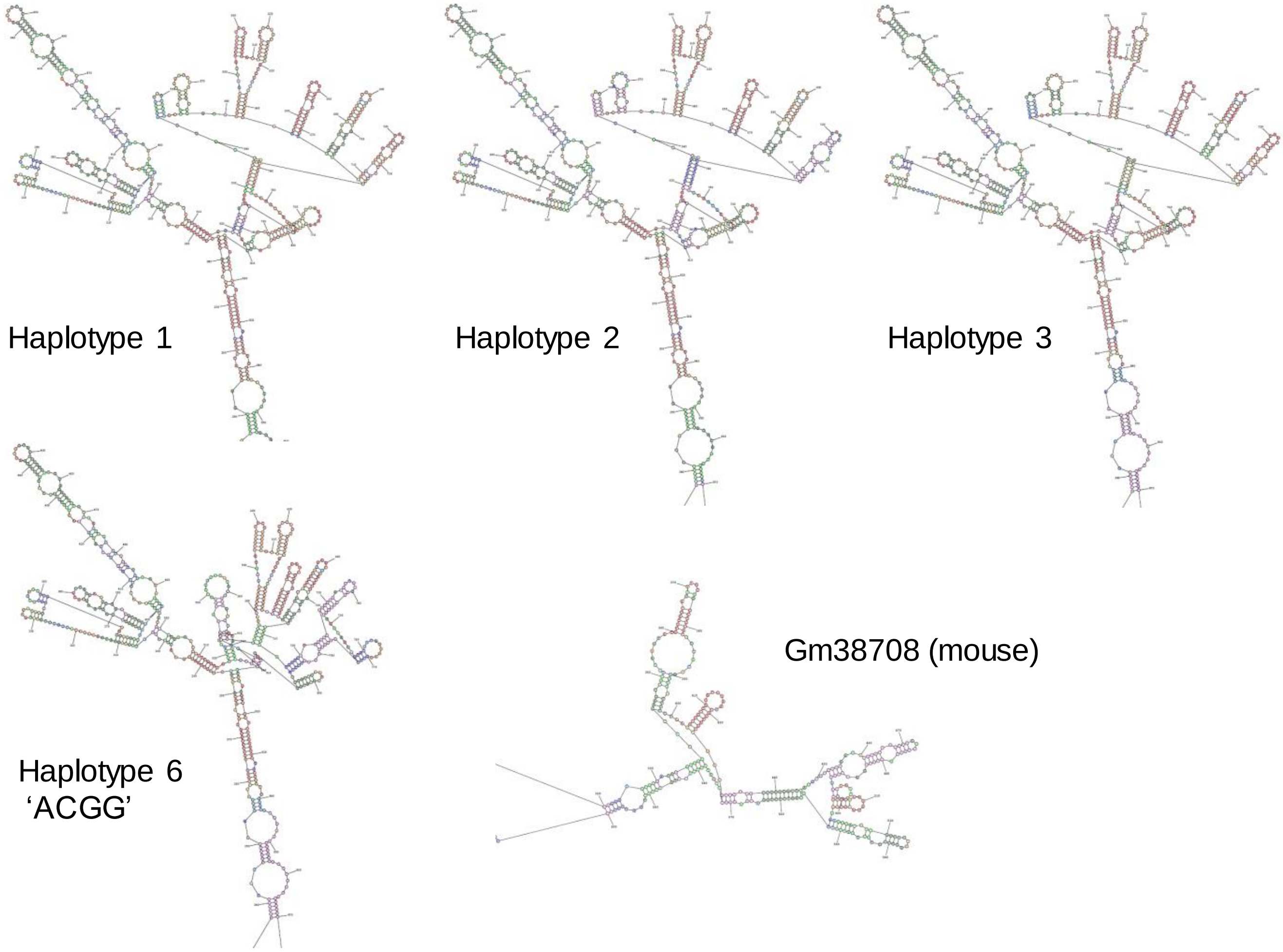

Figure S7. RNA secondary structure predictions for GATA2-AS1 sequence variants and antisense lncRNAs at GATA loci. (A) *In silico* predicted secondary structures (RNAstructure, https://rna.urmc.rochester.edu/RNAstructureWeb/, accessed September 15, 2019)[34] are shown for the common haplotypes for GATA2-AS1 (haplotypes 1-3) as well as the least common haplotype amongst human populations from the 1000 genomes cohort (haplotype 6: 'ACGG'). For comparison, the secondary structure for a mouse ortholog (Gm38708) of human GATA2-AS1 is shown. Note that the predicted secondary structure for haplotype 6 ('ACGG') differs from that of haplotypes 1-3.

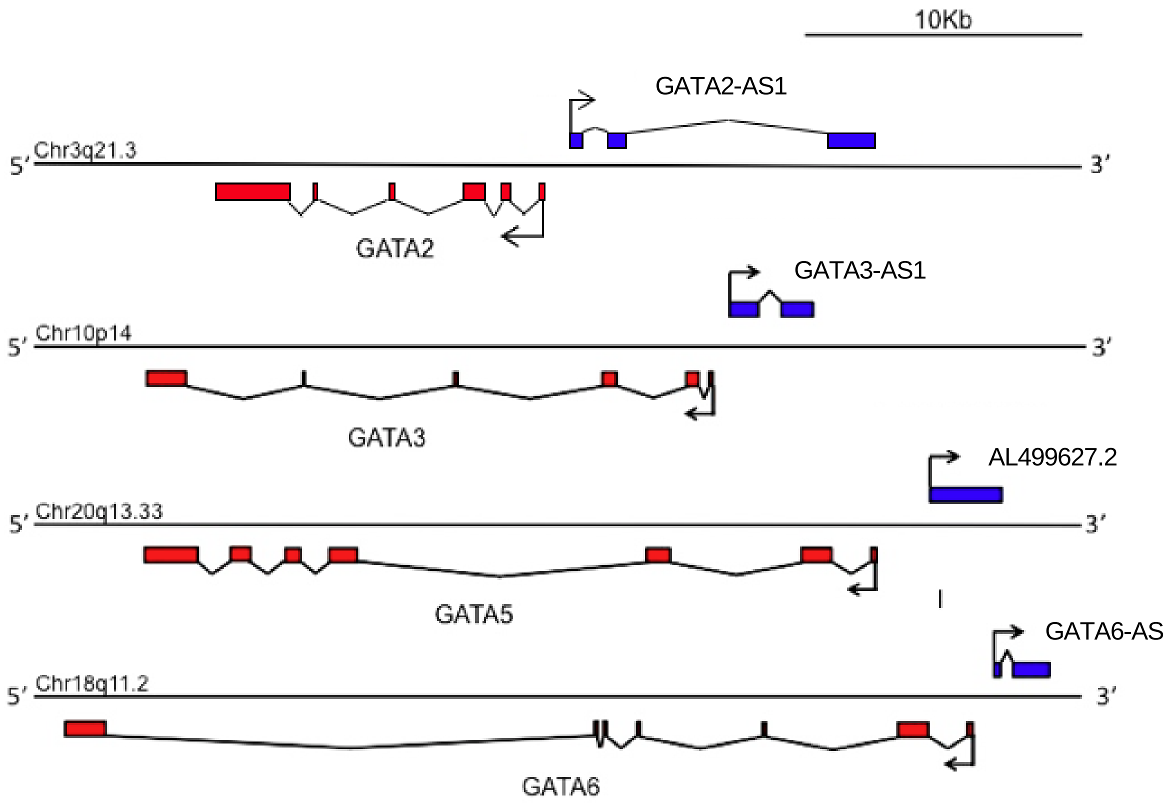

Figure S8. Antisense lncRNAs at GATA loci in humans. Schematic depicting GATA loci in humans with annotated divergent lncRNAs. Divergent lncRNAs are found in both GATA families, GATA123 and GATA456. Antisense lncRNAs are indicated in blue and GATA transcription factors are indicated in red. Scale bar indicates 10kb.

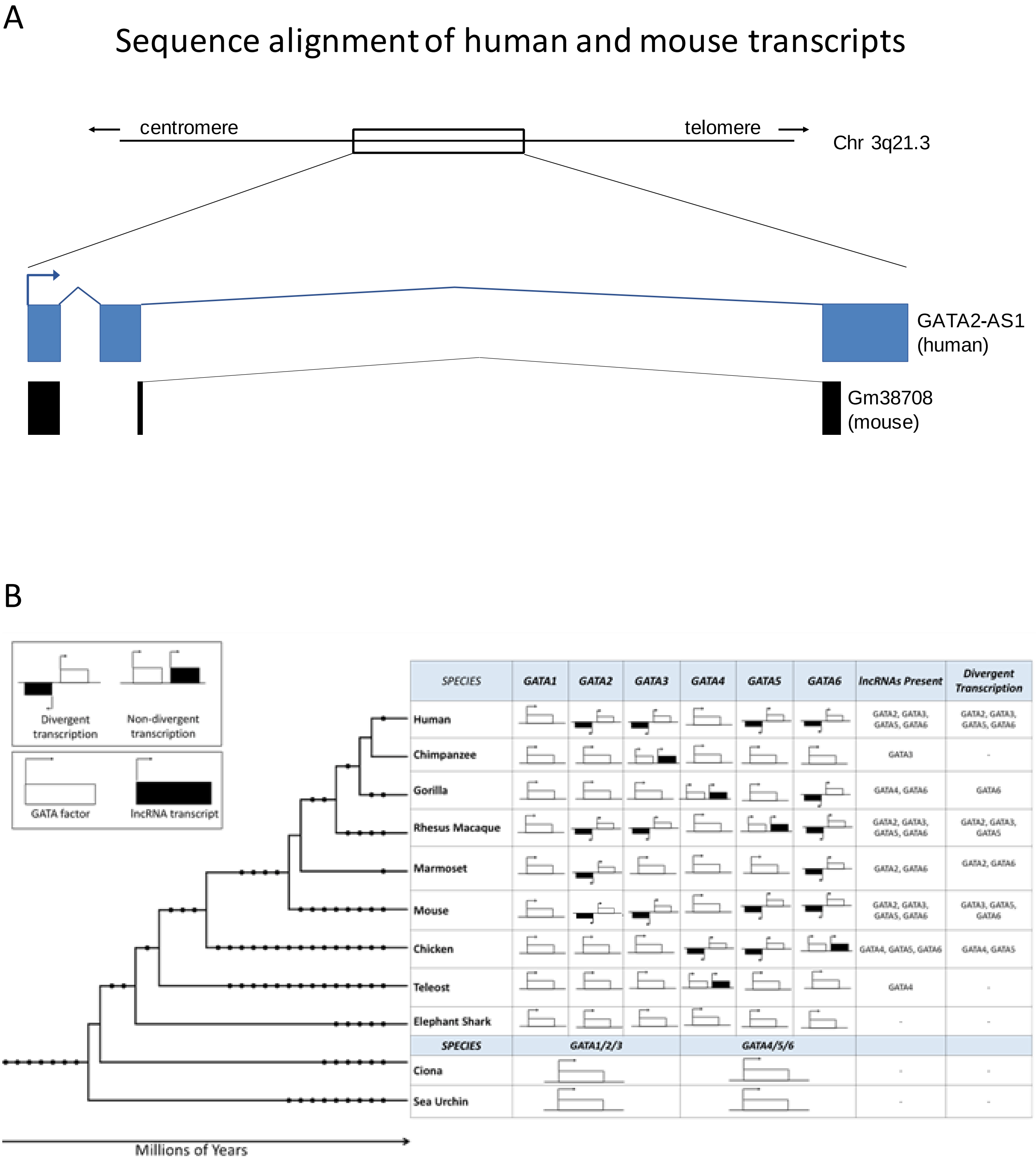

Figure S9. Antisense lncRNAs at GATA loci across species. (A) Conservation of human GATA2-AS1 sequences (ENST00000464242.1) in the mouse Gm38708 (Gm38708-201) from mouse genome GRcm38.p6 downloaded from the Ensembl genome browser July 25, 2019. Human sequences conserved in the mouse are shown in black. Approximately 25.4 % (295/1163 nucleotides from human GATA2-AS1) are conserved in the mouse syntenic transcript. (B) Schematic depicting annotated (RefSeq) sense and antisense lncRNAs in GATA loci across species. The dendrogram indicates approximate evolutionary distance.

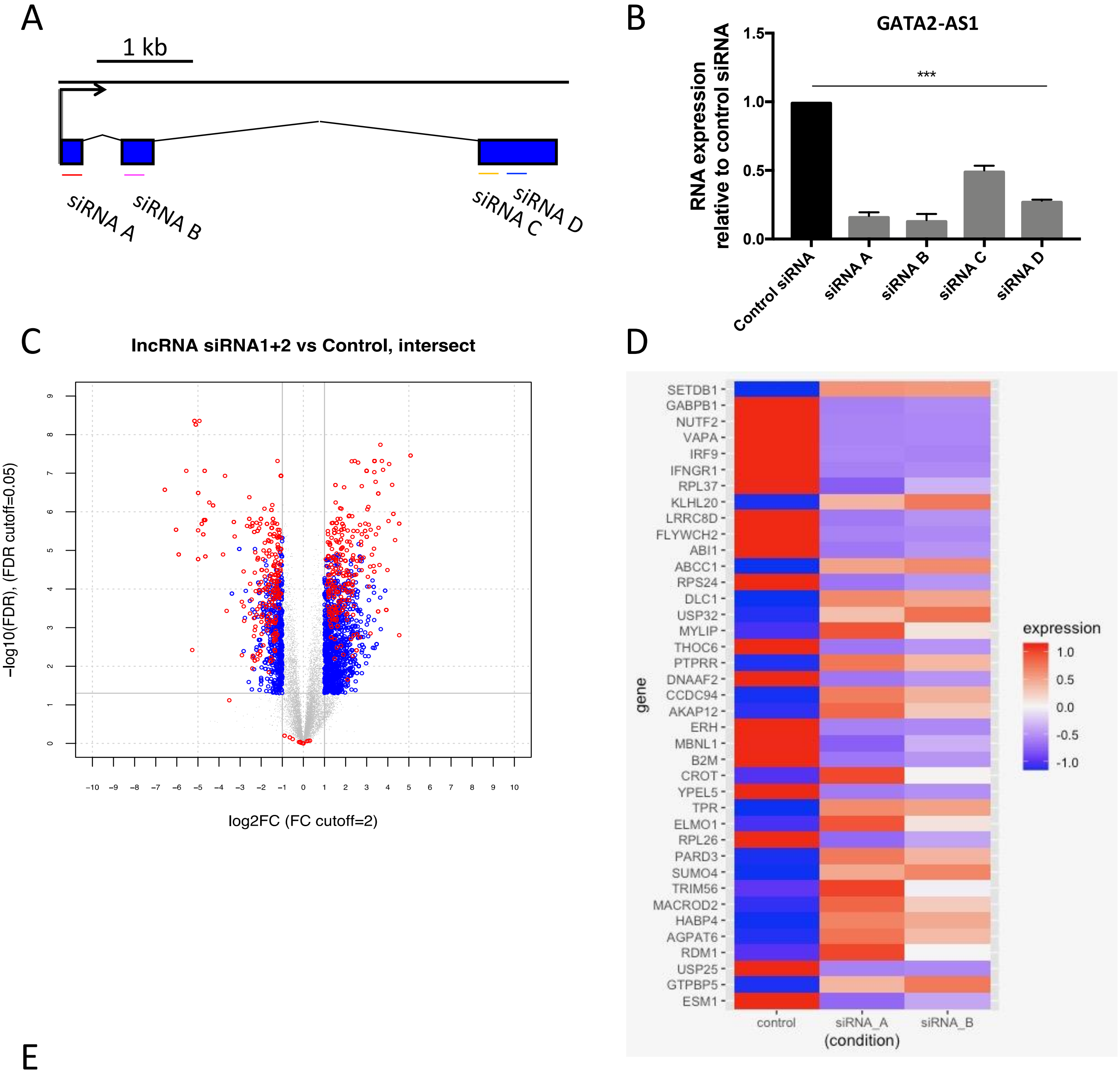

E

GO Term	p-value	# of Genes	% of matched genes in GO Category
**RNA-binding**	2.77E-20	147	9.3%
**Protein binding**	1.77E-13	593	4.7%
**ncRNA metabolic processing**	3.47E-07	50	10.7%
**Nucleoside phosphate binding**	6.08E-07	152	6.4%
**Alternative splicing**	7.25E-04	31	11.0%
**Cellular response to external stimulus**	3.07E-03	29	10.7%
**Cellular response to stress**	3.31E-03	116	6.1%
**Translation**	5.31E-03	52	7.8%
**Angiogenesis**	7.80E-03	16	3.0%

Figure S10. siRNA knockdown of GATA2-AS1. (A) Schematic diagram of GATA2-AS1 with siRNA positions indicated. (B) Knockdown efficiencies of various siRNA sequences. GATA2-AS1 RNA expression is measured by RT-qPCR. siRNA A and siRNA B were the most effective at GATA2-AS1 knockdown. Statistical significance was assessed by 2-sided Student *t* test and * $P < 0.05$. (C) Volcano plot showing microarray analysis of differentially regulated lncRNAs with GATA2-AS1 knockdown. Shown are lncRNAs differentially regulated by both GATA2-AS1 siRNAs. 166 lncRNAs were upregulated and 209 were downregulated. Statistical significance was assessed by Student *t* test with a Benjamini-Hochberg adjusted FDR < 0.05. (D) Heatmap showing microarray data from GATA2-AS1 knockdown. The top 20 upregulated and downregulated genes are shown. Expression level represents z-score of expression for each gene. (E) Gene Ontology (GO) analysis of GATA2-AS1 target genes.

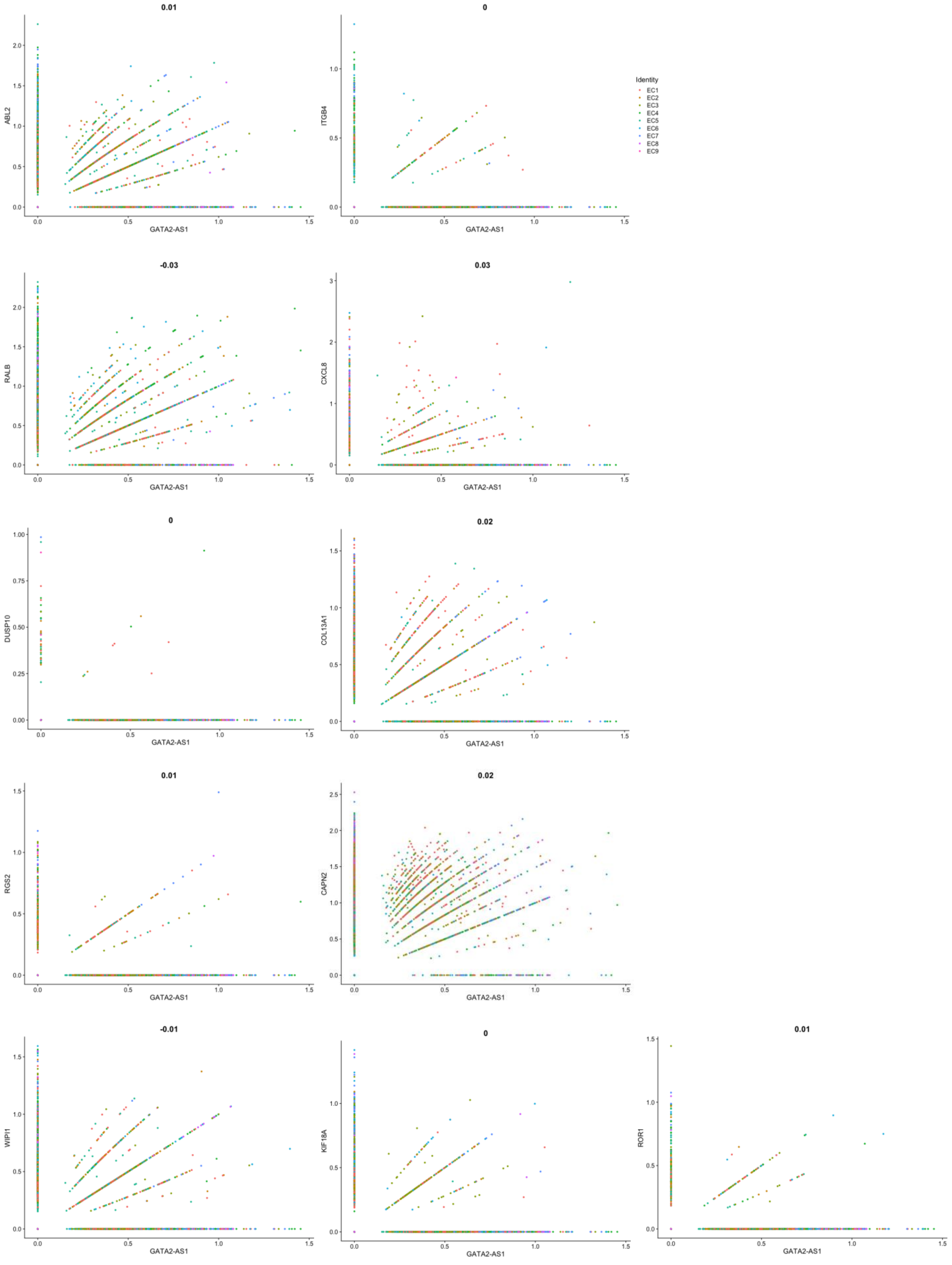

Figure S11. Correlation plots of GATA2-AS1 with GATA2 target mRNAs from passage 3 single-cell RNA-seq.

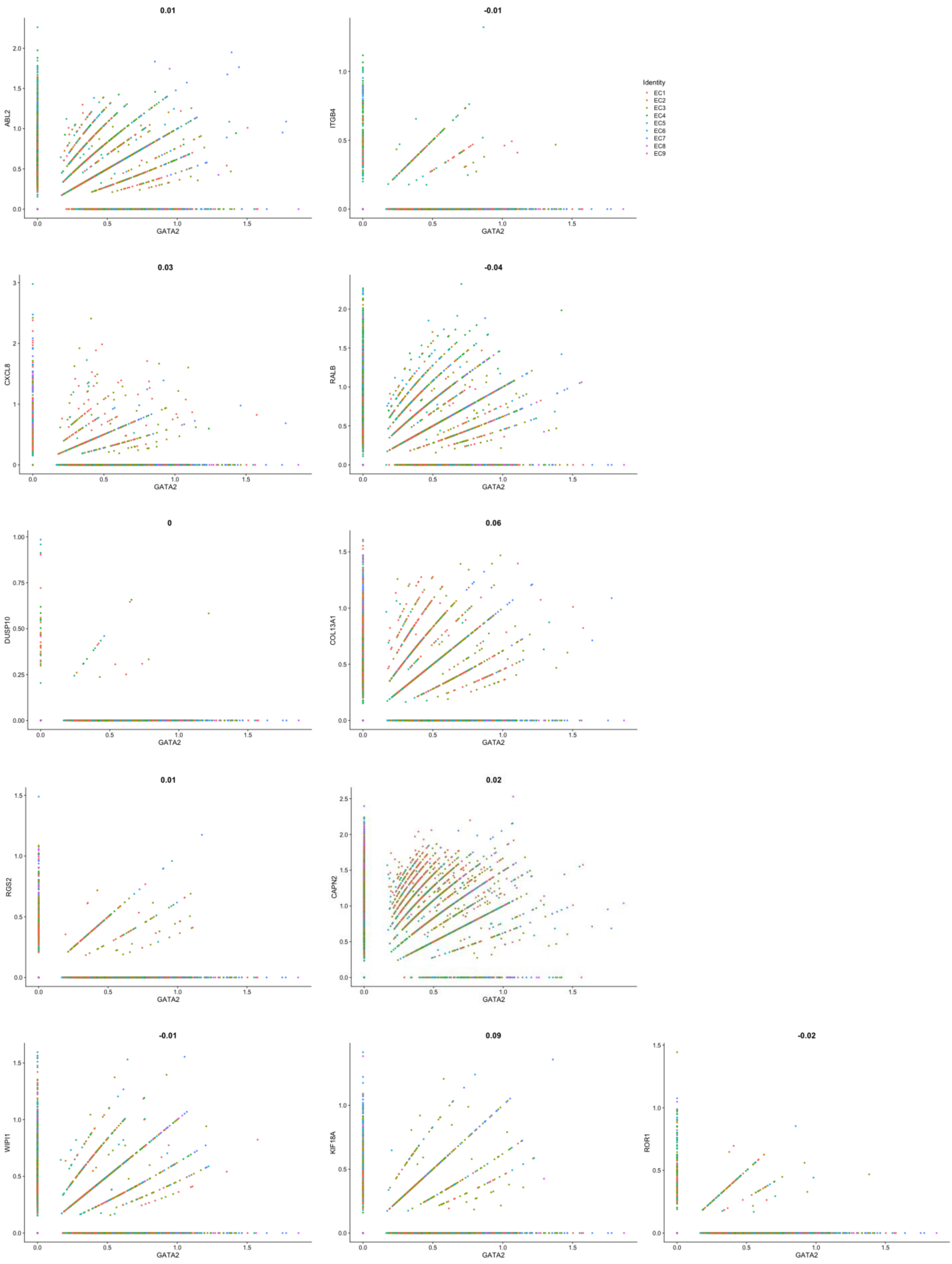

Figure S12. Correlation plots of GATA2 with GATA2-AS1 target mRNAs from passage 3 single-cell RNA-seq.

A

GATA2-AS1 promoter	
**Hypoxia response element (RCGTG)**	Pentamer position relative to TSS
**1**	-648
**2**	-909
**3**	-969

B

GATA2-AS1 promoter	
**Shear stress response element (GAGACC)**	Hexamer position relative to TSS
**1**	-478
**2**	-599
**3**	-944

C

GATA2-AS1 promoter	
**KLF site (CCACCC)**	Hexamer position relative to TSS
**1**	-156
**2**	-845

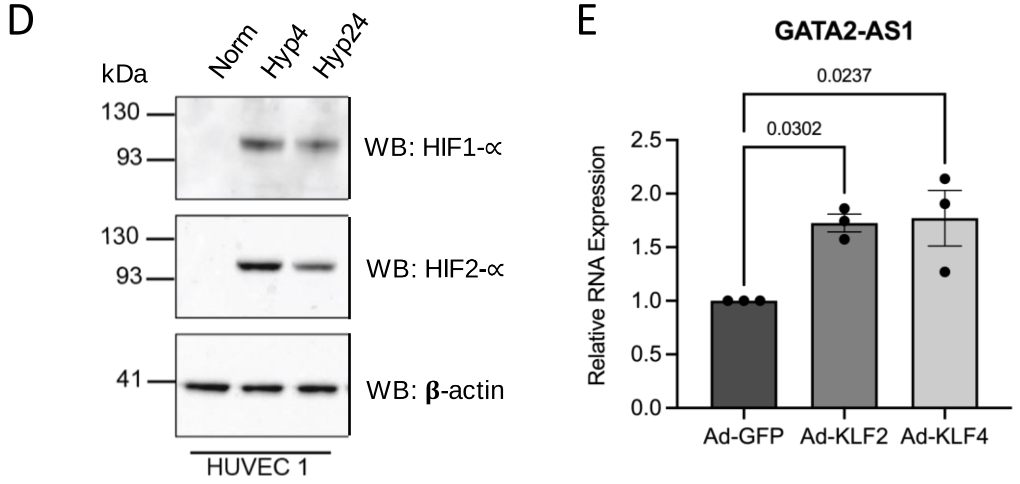

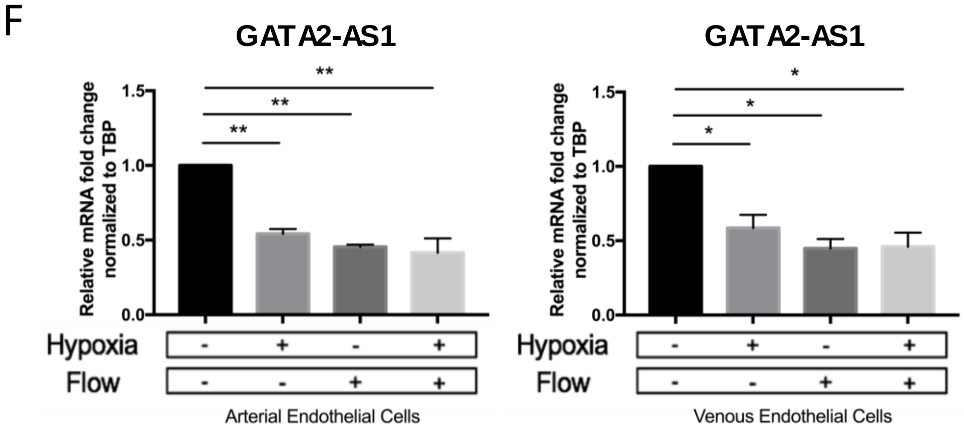

Figure S13. GATA2-AS1 expression with hypoxia and fluid shear stress in endothelial cells. Positions of putative cis-elements relative to the GATA2-AS1 TSS are shown for the hypoxia response element (HRE)(A), the shear stress response element (B), the Kruppel-like factor (KLF) site (C). (D) Representative Western Blots of HIF1-α and HIF2-α with acute (4 hours) and chronic (24 hours hypoxia). β-actin is used a loading control. Both HIF1-α and HIF2-α protein levels increase with hypoxia. (E) Nanostring counts for GATA2-AS1 RNA levels show increase with overexpression of KLF2 and KLF4. (F) RT-qPCR showing GATA2-AS1 expression with laminar flow (10 dynes/cm^2) and/or hypoxia (24 hours) in hPSC-derived arterial-like or venous-like cells[41,42]. In both cell types, GATA2-AS1 RNA expression decreases with both laminar flow and hypoxia. Statistical significance was assessed by One -way ANOVA, SEM, n=3. Normality tested by Shapiro-Wilks test.

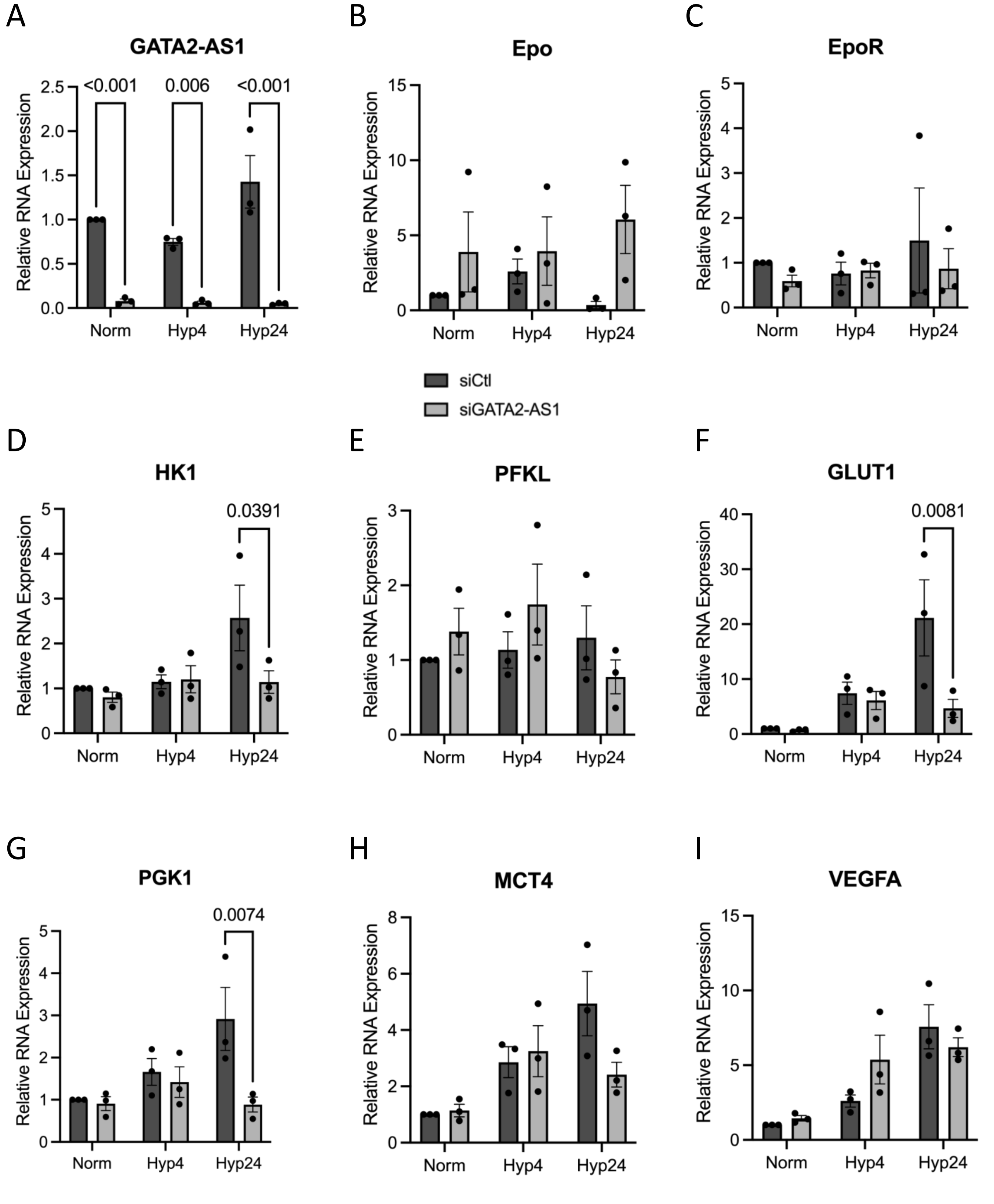

Figure S14. GATA2-AS1 augments hypoxic signalling and upregulation of glycolytic enzymes with hypoxia. (A-I) RT-qPCR of GATA2-AS1, Epo, EpoR, HK1, PFKL, GLUT1, PGK1, MCT4 and VEGFA with GATA2-AS1 knockdown and either acute or chronic hypoxia. RNA expression is normalized to “normoxia” and “siRNA control” conditions to show changes in RNA levels with hypoxia. Statistical significance was assessed by Two-way ANOVA, SEM, n=3. Normality tested by Shapiro-Wilks test.

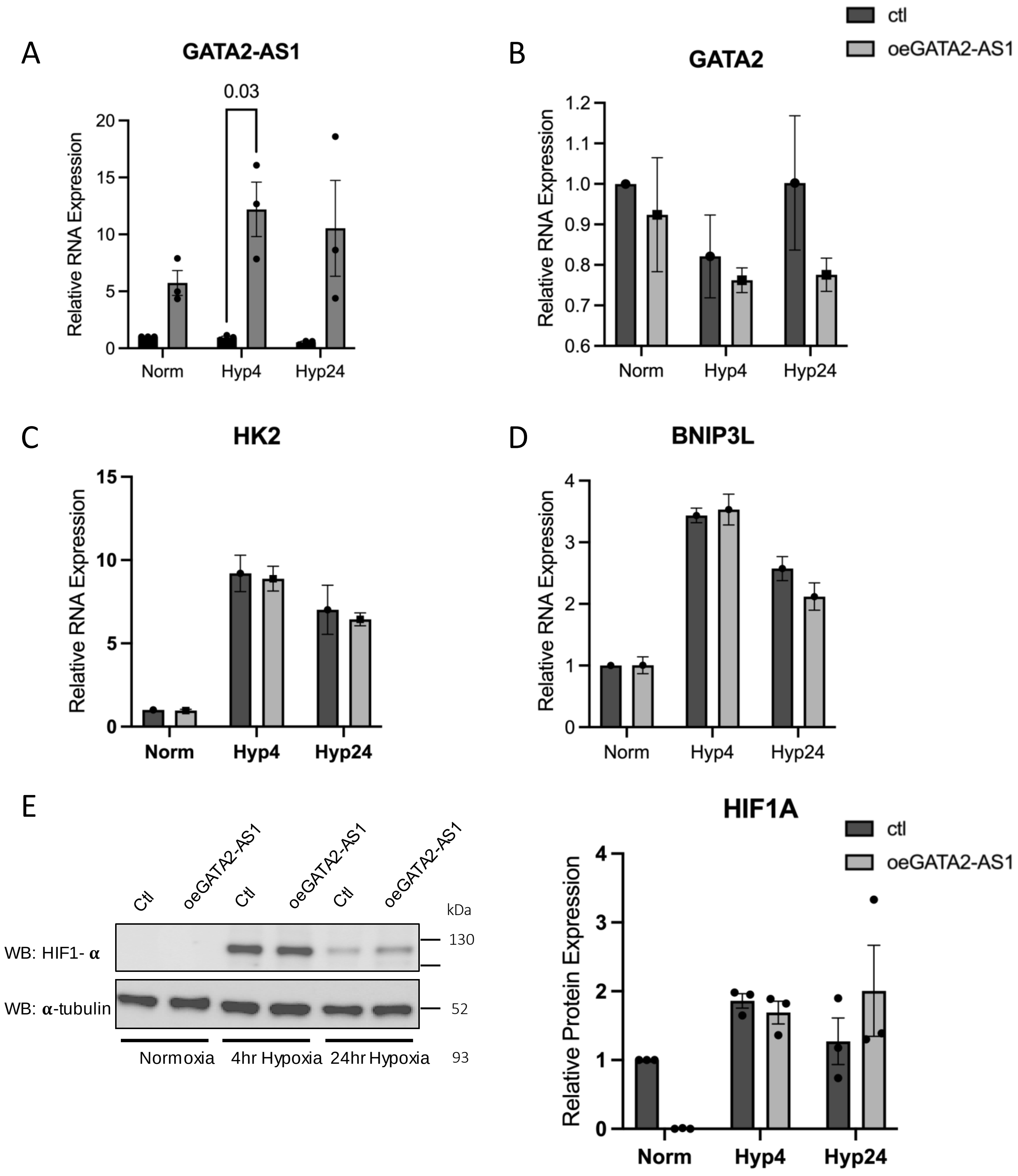

Figure S15. Overexpression of GATA2-AS1 in hypoxia. (A) RT-qPCR of GATA2-AS1 showing overexpression of GATA2-AS1 vs control lentivirus in normoxia (Norm), 4 hours hypoxia (Hyp4) and 24 hours hypoxia (Hyp24). (B-D) RT-qPCR of GATA2, HK2 and BNIP3L with overexpression of GATA2-AS1 vs control lentivirus in Norm, Hyp4, and Hyp24. There is no statistically significant change in GATA2, HK2, or BNIP3L with GATA2-AS1 overexpression. (E) Western Blot of HIF1-α with overexpression of GATA2-AS1 vs control lentivirus in Norm, Hyp4, and Hyp24. α-tubulin is shown as a loading control. Representative blot is shown on the left and quantitation of multiple blots is shown on the right. There is a small but variable increase in HIF1-α protein levels with 24 hours hypoxia with GATA2-AS1 overexpression. Statistical significance was assessed by Two-way ANOVA, SEM, n=3. Normality tested by Shapiro-Wilks test.

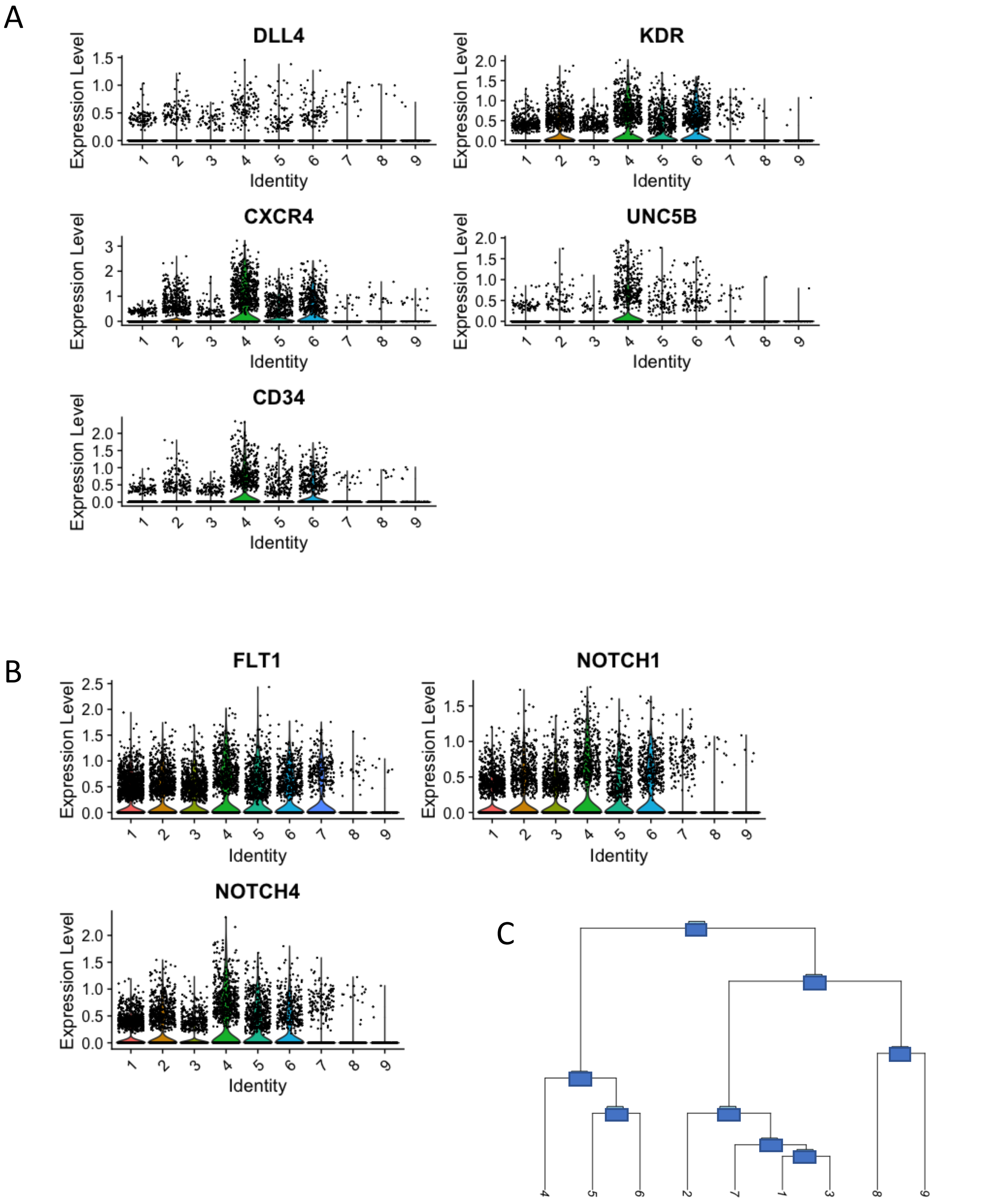

Figure S16. Single-cell RNA sequencing reveals expression patterns of "tip cell" and "stalk cell" genes. (A) "Tip cell" genes show greatest expression in clusters 4-6. (B) "Stalk cell" genes show expression across clusters 1-6. (C) Dendrogram showing clusters as a tree. Note that clusters 4-6 group together on a separate branch from the other clusters.

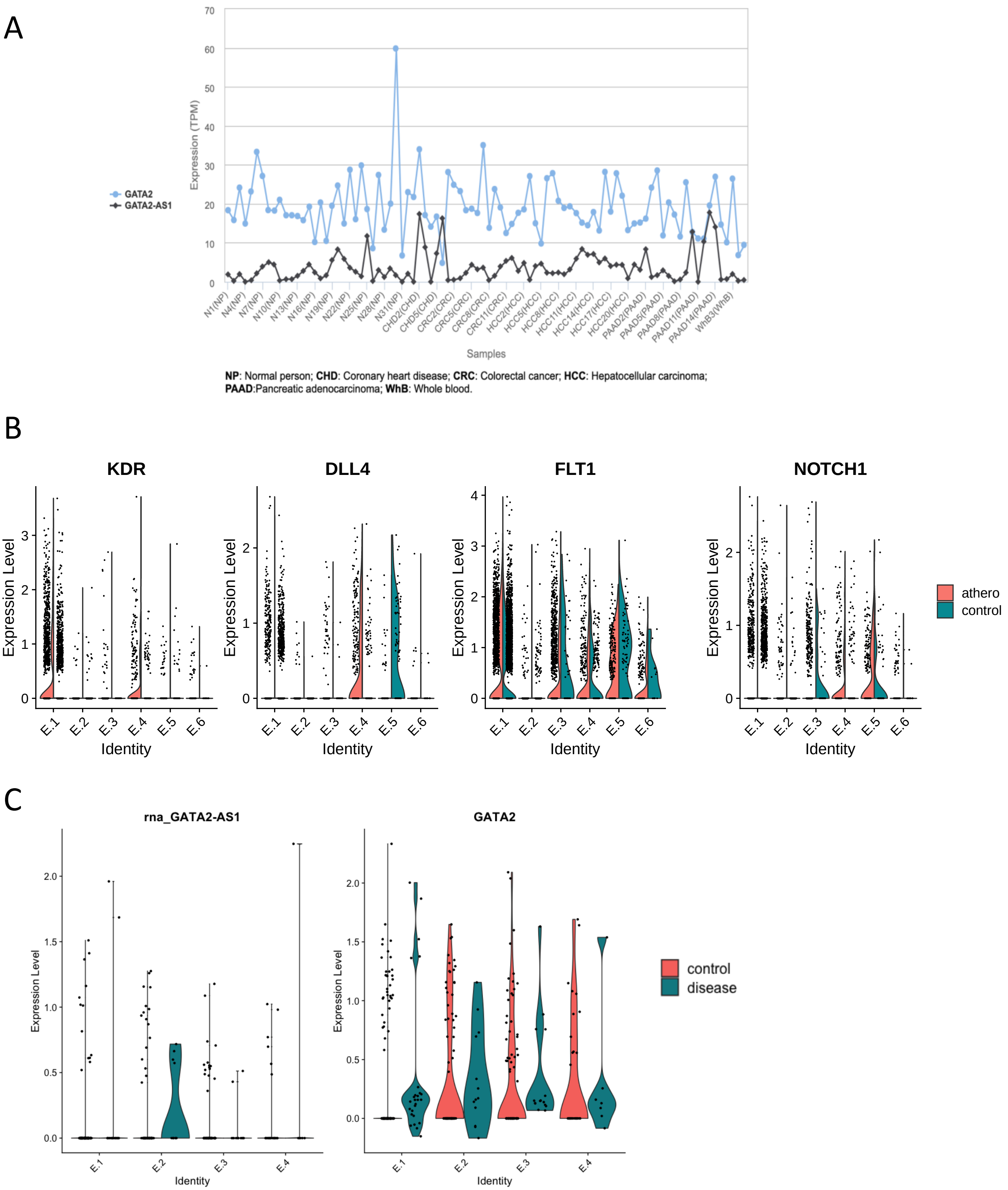

Figure S17. Gene expression in human disease samples. (A) Individual level data from exoRBase v1 (http://www.exorbase.org accessed June 23, 2021) show expression of GATA2-AS1 (black) and GATA2 (blue) RNA in exosomes from healthy control and disease samples. (B) Violin plots from scRNAseq of human atherosclerosis tissue shows expression of "tip cell" genes (KDR, DLL4) and "stalk cell" genes (FLT1, NOTCH1) in endothelial cell clusters (E.1 – E.6). There is differential expression of both "tip cell" and "stalk cell" genes between control tissue (control) and atherosclerosis core tissue (athero). Athero = atherosclerosis core, control = proximal adjacent tissue from the carotid artery. (C) Violin plots from scRNAseq of human aortic aneurysm tissue shows expression of GATA2-AS1 and GATA2 in human aortic endothelial cells as perturbed in disease.

Supplemental Video 1 – Time lapse video of spheroid sprouting under normoxic conditions for 24h.
Supplemental Video 2 - Time lapse video of spheroid sprouting under normoxic conditions for 24h.
